# Evolution of beak morphology in the Ground Tit revealed by comparative transcriptomics

**DOI:** 10.1186/s12983-017-0245-6

**Published:** 2017-12-21

**Authors:** Yalin Cheng, Bin Gao, Haitao Wang, Naijian Han, Shimiao Shao, Shaoyuan Wu, Gang Song, Yong E. Zhang, Xiaojia Zhu, Xin Lu, Yanhua Qu, Fumin Lei

**Affiliations:** 1 Key Laboratory of Zoological Systematics and Evolution, Institute of Zoology, Chinese Academy of Sciences, Beijing, 100101 China; 2 School of Life Sciences, Northeast Normal University, Changchun, 130024 China; 3 School of Life Sciences, Jiangsu Normal University, Xuzhou, 221116 China; 4 University of Chinese Academy of Sciences, Beijing, 100049 China; 5 Department of Ecology, College of Life Sciences, Institute for Advanced Studies, Wuhan University, Wuhan, 430072 China

**Keywords:** *Parus humilis*, Beak, Morphology, Transcriptomics, Osteoblasts, Osteoclasts

## Abstract

**Background:**

Beak morphology exhibits considerable adaptive plasticity in birds, which results in highly varied or specialized forms in response to variations in ecology and life history. As the only parid species endemic to the Qinghai-Tibet Plateau, the Ground Tit (*Parus humilis*) has evolved a distinctly long and curved beak from other parids. An integration of morphometrics, phylogenetics, transcriptomics and embryology allows us to address the evolutionary and developmental mechanisms of the adaptive beak structure observed in the Ground Tit.

**Results:**

A morphometric approach quantified that the Ground Tit has a comparatively longer and more decurved upper beaks than other parids. We estimated that the ancestor of the Ground Tit likely had a short straight upper beak similar to most current recognized parid species using an ancestral state reconstruction. This morphological specialization is considered an adaptation to its ground-oriented behavior on the high plateau. To identify genetic mechanisms behind this adaptive change, a comparative transcriptomic analysis was applied between the Ground Tit and its closely related species, the Great Tit (*Parus major*). We detected that 623 genes were significantly differentially expressed in embryonic upper beaks between the two species, 17 of which were functionally annotated to correlate with bone development and morphogenesis, although genes related to bone development were not found to undergo accelerated evolution in the Ground Tit. RT-qPCR validation confirmed differential expression of five out of eight genes that were selected from the 17 genes. Subsequent functional assays in chicken embryos demonstrated that two of these genes, *FGF13* and *ITGB3*, may affect beak morphology by modulating levels of osteoblasts and osteoclasts.

**Conclusions:**

Our results provide preliminary evidence that development of the long decurved beak of the Ground Tit is likely regulated by transcriptional activities of multiple genes coordinating osteoblasts and osteoclasts. The integration of multiple approaches employed here sheds light on ecological and genetic mechanisms in the evolution of avian morphology.

**Electronic supplementary material:**

The online version of this article (10.1186/s12983-017-0245-6) contains supplementary material, which is available to authorized users.

## Background

The avian beak is a highly evolvable structure. The malleability of beak morphology permits birds to adapt, sometimes rapidly, to diverse ecological niches [1]. Various interrelated ecological and behavioral aspects of diet and feeding, such as local and seasonal food availability, dietary preference, and acquisition and manipulation of food and water (e.g., food crushing, grasping, drinking and probing), likely act as the major selective forces on beak morphology [2]. Other factors in addition to diet, such as sexual selection and thermal regulation, may also play a role in shaping variation in beak morphology [3, 4]. Understanding the underlying genetic mechanisms involved in beak development is important for understanding the ultimate evolutionary causes of beak diversity in birds and the nature of evolutionary adaptation [5].

Studies of the genetic mechanisms underlying variation in beak morphology have identified multiple genes responsible for the development of beak morphology in birds. For example, *BMP4* has been found to play a role in regulating both the width and depth of the prenasal cartilage (pnc) in the frontal nasal mass (FNM) of both Darwin’s finches and chickens [6, 7]. The differential expression of other genes, such as *CALM1*, *IHH*, *DKK3*, *TGFBR2* and *CTNNB1*, is responsible for regulating the length and width of the pnc and premaxillary bone (pmx) in birds [8–10]. Two recent genomic analyses on Darwin’s finches revealed that variations of *ALX1* and *HMGA2* are associated with beak shape [11] and beak size [12], respectively. These results show that the development of beak morphology is regulated by a complex genetic system that involves multiple genes. It is very similar to the craniofacial development across vertebrates, which is controlled by the conserved and complicated multi-gene pathways [13–15].

Traditionally, the Ground Tit (*Parus humilis*) was considered to be a member of the family Corvidae based on its phenotypic similarities with ground jays of the genus *Podoces* (e.g., the long and curved beak) [16, 17]. However, phylogenetic studies placed the Ground Tit within the family Paridae and supported that its jay-like decurved beak is homoplasious [18, 19]. The Ground Tit is the only parid species that lives in the high altitude steppes and meadows of the Qinghai-Tibet Plateau, where it resides between 3100 and 5500 m. Other Paridae members are distributed in forested habitats in low altitudinal areas across Eurasia, North America and Africa [20]. In response to the selective pressure imposed by the foraging pattern and burrow-nesting habits in the sparsely vegetated, treeless and high altitude environment [21, 22], the Ground Tit has evolved a unique beak morphology that is distinct from the short and straight beak of all other parids [23].

To explore the evolutionary and developmental mechanisms of the adaptive beak structure observed in the Ground Tit, we firstly employed linear and geometric analyses to quantify variations in beak morphology between the Ground Tit and other parids. We then identified potential candidate genes contributing to beak development in the Ground Tit by comparative transcription between the Ground Tit and the Great Tit (*Parus major*). They are closely related species with a divergence time of approximately 7.7-9.9 million years [19]. We further tested the function of the candidate genes in chicken embryos with recombinant proteins. Our results shed new light on the evolution of beak morphology in a non-model species and possibly in other extensive bird species as well.

## Results

### A derived long decurved beak in the Ground Tit

**Fig. 1:**
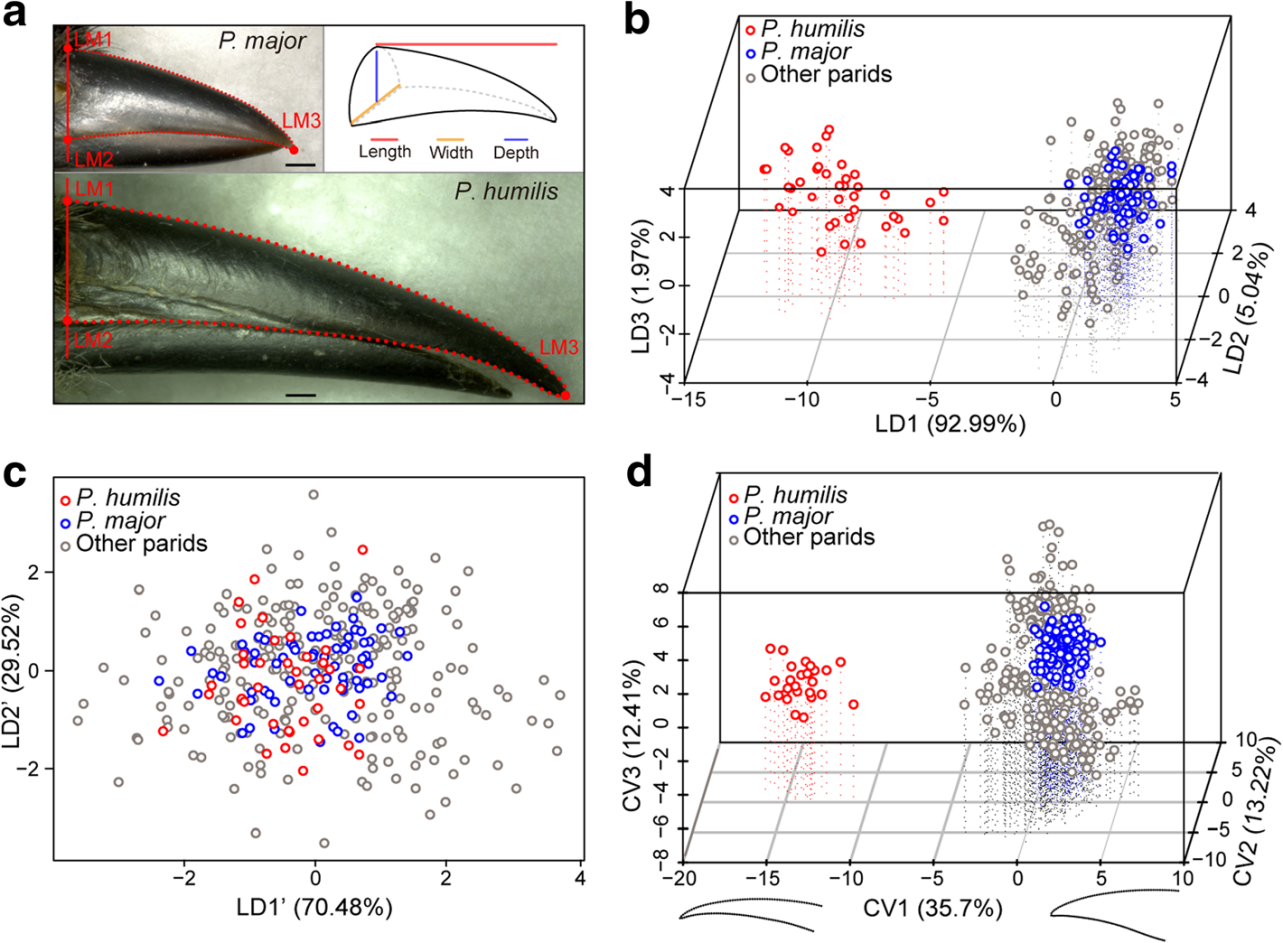
Morphometric analyses for beak size and beak shape of 13 parid species. **a.** The rule of measurement for the size (length, width and depth) of upper beaks and the selection of landmarks and semi-landmarks on upper beaks. Scale bars are equal to 1 mm. **b.** LDA for all three size parameters. The 3D plot is based on three linear discriminant functions: LD1, LD2 and LD3. Ground Tits are clearly separated from other tits. **c.** LDA for beak width and depth. Ground Tits overlap with other parids. **d.** CVA of beak shape based on Procrustes coordinates. The first three CV components, CV1, CV2 and CV3, cumulatively account for 61.33% of total shape variation. Ground Tits are clearly separated from other parids. CV1 predicted the variations in beak shape between the Ground Tit and all other parid species. CV2 and CV3 predicted differences among other parids (see Additional file 1: Figure S1b)

Length, width and depth of upper beaks from 349 skin specimens were measured for linear analysis (Fig. 1a). There were no significant phylogenetic signals in three size characteristics (beak length, width, depth) of the parids (see Additional file 1: Table S1), suggesting that beak size evolved independently from phylogeny. Both Hotelling-Lawley’s test (Hotelling-Lawley trace = 20.51, *F*_36, 998_ = 189.54, *P* < 0.001) and linear discriminant analysis (LDA) showed that beak size in the Ground Tit is significantly different from that in all other parids (Fig. 1b; see Additional file 1: Tables S2 and S3). However, the LDA without beak length could not discriminate Ground Tits from other parids (Fig. 1c; see Additional file 1: Tables S2 and S3), which indicated that beak length is a better discriminant variable than beak width and depth. An LDA with only beak length further showed that the length is the major variation in beak size between the Ground tit and other parids (see Additional file 1: Table S3, Figure S1a), which is similar to previous results based on a subsection of current dataset [23] (see Additional file 2).

A total of 352 upper beak profiles collected from our previous work [23] and newly photographed images were used to perform geometric analysis (Fig. 1a, see Additional file 3). We did not find significant phylogenetic signals in Procrustes coordinates (shape, *P* = 0.802) and centroid size (size, *P* = 0.431), indicating that evolution of beak shape was also phylogenetically independent. Multivariate regression detected no significant allometry between shape and size (*P* = 0.064) even though 49.04% of total interspecific variation in shape was predicted by size variation. Procrustes ANOVA on Procrustes coordinates showed significant interspecific difference in beak shape (*F*_2808, 79,326_ = 63.14, *P* < 0.001). Discriminant function analysis (DFA) and canonical variate analysis (CVA) clearly distinguished Ground Tits from all other parids (Fig. 1d, see Additional file 1: Tables S4 and S5). Although CV1 explained only 35.70% of total variation in beak shape across 13 species (see Additional file 1: Table S6), the variation in CV1 represented differences between the Ground Tit and other parids (Fig. 1d, see Additional file 1: Figure S1b), which was indicated by the change of beak shape from a blunt, robust and straight to a pointed, slender and decurved one in CV1 axis (Fig. 1d).

**Fig. 2:**
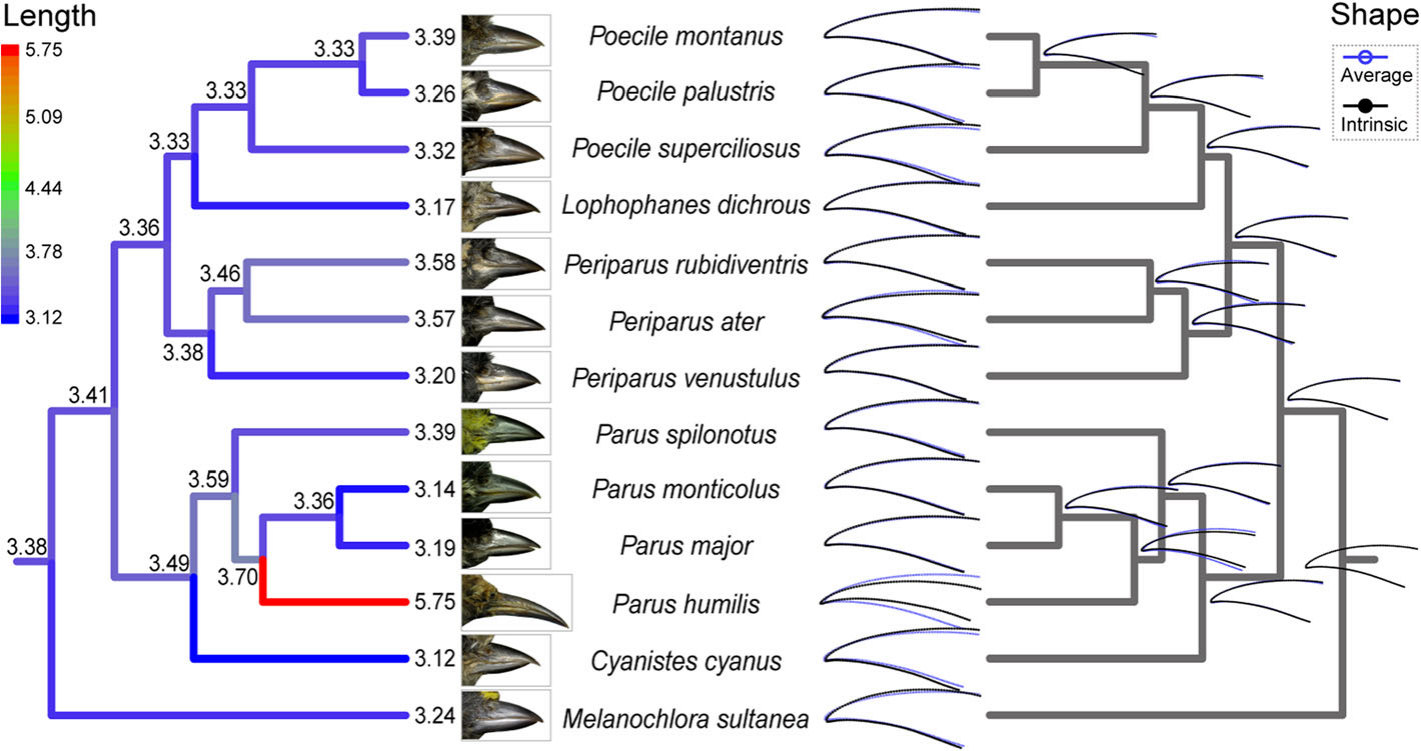
Ancestral states reconstruction for beak length and shape of 13 parid species. The node labels in the length tree (left) are the estimated length of ancestral beaks. The beak profiles corresponding to the nodes in the shape tree (right) are the estimated ancestral beak shapes. The blue profiles are the average shape, while the black profiles are the real shape. The images of beaks were taken from specimen of National Zoological Museum of the Institute of Zoology, Chinese Academy of Sciences

An ancestral state reconstruction of beak morphology estimated that both the ancestor of Ground Tits and the common ancestor of the parids had beak morphology similar to that of the majority of extant taxa within the Paridae (Fig. 2). The ancestral beak of Ground Tits was medium sized and straight shaped, resulting in a short and relatively straight beak compared to extant Ground Tits (Fig. 2; see Additional file 1: Figure S2). Similarly, the putative common ancestor of the parids exhibited a medium sized and straight shaped beak.

### Coding sequence in beak-related genes underwent unaccelerated evolution

**Fig. 3:**
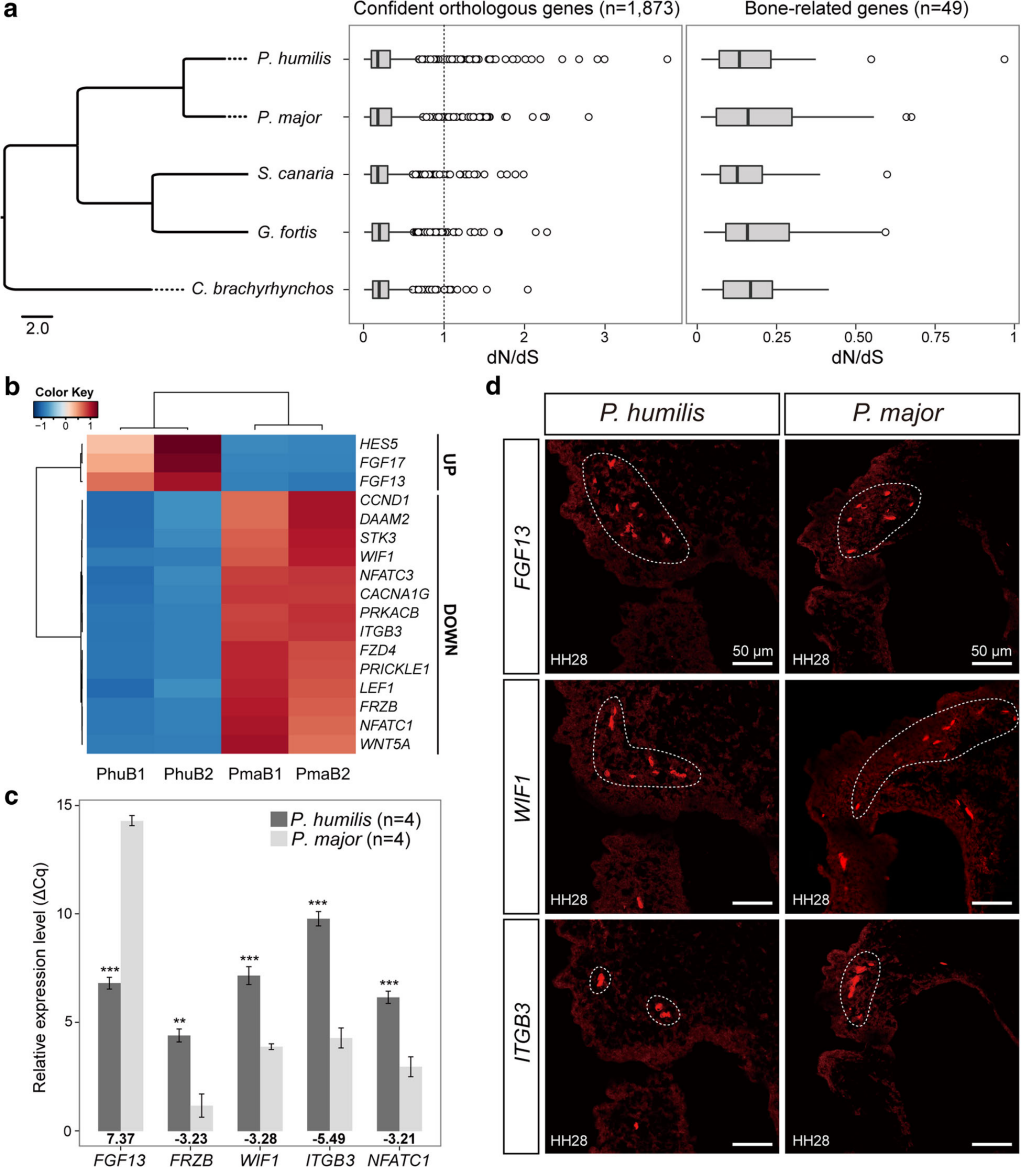
Effects of changes in coding sequence and gene expression on beak morphology. **a.** Evolutionary analysis showed no accelerated evolution in coding sequences of the Ground Tit. The left phylogenetic tree was constructed using BEAST. We compared dN/dS ratios between each lineage for 1873 retained genes (middle box plot) and 49 bone-morphogenesis-related genes (right box plot). **b.** RNA-Seq analyses revealed expression patterns of the 17 candidate genes that were related to bone development. These genes can be divided into up- and down-regulated groups. PhuB1, replicate 1 of Ground Tit beak; PhuB2, replicate 2 of Ground Tit beak; PmaB1, replicate 1 of Great Tit beak; PmaB2, replicate 2 of Great Tit beak. **c.** RT-qPCR experiments confirmed the significant differences in expression of *FGF13*, *FRZB*, *WIF1*, *ITGB3* and *NFATC1* between Ground Tits and Great Tits. The fold-change values of these genes are shown under the bars. ***, *P* < 0.001. **, *P* < 0.01. **d.** In situ hybridization assays for spatial expression patterns of *FGF13*, *WIF1* and *ITGB3* on sections of upper beaks. A white dotted box circles the expression domains

A total of 6310 orthologous genes among the Ground Tit, Great Tit, Medium Ground Finch (*Geospiza fortis*), Atlantic Canary (*Serinus canaria*) and American Crow (*Corvus brachyrhynchos*) were identified for phylogenomic analysis. After removing genes with errors and saturated mutations, 1873 genes were retained for subsequent analyses. For all 1873 genes, the mean dN/dS ratio of the Ground Tit was higher than that of other lineages, but these differences were not statistically significant (Fig. 3a; see Additional file 1: Tables S7 and S8). And the genes with dN/dS > 1 in the Ground Tit were found to participate in repairing damaged DNA and responding to stress instead of dedicating to bone morphogenesis (see Additional file 1: Table S9).

When we functionally annotated the 1873 retained genes, 49 genes that were identified to contribute to bone development and remodeling (see Additional file 1: Table S10) did not show statistically significant differences in dN/dS ratios between the Ground Tit and the other four lineages (Fig. 3a; see Additional file 1: Tables S7 and S8), although they have quite different beak morphotypes. Moreover, none of the 49 genes in the Ground Tit was assigned both an elevated dN/dS ratio and a significant likelihood value (see Additional file 1: Figure S3).

### Gene expression differentiation between two beak morph types of the Ground Tit and the Great Tit

Transcriptomes were sequenced for four embryonic upper beaks at stages 28/29 (see Additional file 1: Figure S4) from two Ground Tits and two Great Tits (see Additional file 1: Figure S5). Approximately 19.53-19.99 and 22.88-25.30 million clean reads were mapped to the Ground Tit [19] and the Great Tit [24] genomes, respectively (see Additional file 1: Figure S6a). Expressions of 9217 orthologous genes displayed considerably higher variation between species than that within species (see Additional file 1: Figure S6b). DESeq identified 2730 genes with statistically significant differences in expression between Ground Tits and Great Tits, edgeR identified 3183 significantly different genes, and NOISeq identified 1262 (see Additional file 1: Figure S7a-c). By combining results of above three approaches (see *Methods*), we defined 623 genes to be significantly differentially expressed, including 148 up-regulated and 475 down-regulated genes in the Ground Tit relative to the Great Tit (see Additional file 1: Figure S7d; see Additional file 4).

Although KEGG enrichment analysis of differentially expressed genes did not identify pathways with significantly FDR-adjusted *P*-values, we found several conserved pathways to be likely relevant to beak or face morphogenesis [25], including Wnt (ko04310), MAPK/FGF (ko04010), Osteoclast differentiation (OCD) (ko04380), Calcium (ko04020) and Notch signaling pathways (ko04330) (see Additional file 1: Table S11, Figure S8a). These pathways involved 26 genes, 17 of which were functionally annotated to be related to bone development and morphogenesis, including 3 up-regulated and 14 down-regulated genes (Table 1 and Fig. 3b, see Additional file 1: Table S12). The function categories of these genes were mostly clustered in the top 20 GO terms, such as cartilage development (GO:0051216), anatomical structure morphogenesis (GO:0009653), connective tissue development (GO:0061448) and skeletal system development (GO:0001501) (see Additional file 1: Figure S8b).

**Table 1:** Descriptions and expression patterns of 17 differentially expressed genes that probably correlate with beak morphology

Pathways	Genes	Descriptions	TPM		NOISeq		RT-qPCR	
			*P. humilis*	*P. major*	Log2FC	*P*-adj	Log2FC	*P*
Wnt	*WNT5A*	Protein Wnt-5a	48.06	148.61	−1.63	0.00E+00	–	–
	*PRICKLE1*	Prickle-like protein 1	14.86	55.20	−1.89	2.75E-06	−0.17	1.10E-01
	WIF1*†	Wnt inhibitory factor 1	7.28	57.05	−2.97	8.87E-06	−3.28	5.30E-06
	*NFATC3*	Nuclear factor of activated T-cells, cytoplasmic 3	8.98	39.30	−2.13	9.47E-05	–	–
	*FZD4*	Frizzled-4	16.67	89.95	−2.43	1.54E-04	–	–
	*LEF1*	Lymphoid enhancer-binding factor 1	60.50	143.01	−1.24	1.77E-04	–	–
	FRZB*	Secreted frizzled-related protein 3	71.32	150.58	−1.08	3.47E-04	−3.23	4.12E-05
	*DAAM2*	Disheveled-associated activator of morphogenesis 2	2.48	15.04	−2.60	3.47E-04	–	–
	*CCND1*	G1/S-specific cyclin-D1	24.35	69.13	−1.51	4.18E-04	–	–
	*PRKACB*	cAMP-dependent protein kinase catalytic subunit beta	34.22	73.42	−1.10	4.97E-04	–	–
MAPK/FGF	*FGF17*	Fibroblast growth factor 17	45.91	0.38	6.91	0.00E + 00	0.50	4.18E-01
	FGF13*†	Fibroblast growth factor 13	11.33	3.71	1.61	4.21E-04	7.37	1.23E-08
	*STK3*	Serine/threonine-protein kinase 38	11.70	40.72	−1.80	4.64E-04	–	–
OCD	NFATC1*	Nuclear factor of activated T-cells, cytoplasmic 1	5.82	39.32	−2.76	1.60E-08	−3.21	2.16E-05
	ITGB3*†	Integrin beta-3	0.76	6.43	−3.08	8.57E-05	−5.49	1.21E-06
Notch	*HES5*	Transcription factor HES-5	117.76	17.23	2.77	3.11E-04	0.22	6.83E-01
Calcium	*CACNA1G*	Voltage-dependent T-type calcium channel subunit alpha-1G	4.28	14.60	−1.77	5.79E-04	–	–

RNA-Seq analyzed expression patterns that were subsequently validated by RT-qPCR, in situ hybridization and functional experiments. The expression levels and patterns are shown with the results of NOISeq. Please see results of DESeq and edgeR in Additional file 2: Table S11. TPM (transcript per million) represents the expression level. Log2FC is the transformation of the fold change in expression levels. *P*-adj is the adjusted *P*-value using FDR, which is considered equivalent to 1 − q_NOISeq_ in NOISeq. *P* in the last column is produced by student’s t-test for the relative expression of both species from RT-qPCR. Nine non-validated genes in RT-qPCR are filled with “--” in last two columns. Five confirmed genes by RT-qPCR are marked with “*”. “†” marks the three genes assayed by in situ hybridization. Two proved genes by functional experiments are bolded

Eight genes were selected from the 17 genes (see *Methods*) to be validated for their observed expression levels and patterns in embryonic upper beaks at stages 28/29 using reverse transcription quantitative PCR (RT-qPCR). RT-qPCR validation confirmed five out of the eight genes showing significant differences in expression in the upper beaks of Ground Tits relative to that in Great Tits, including *FGF13* in FGF signaling pathway, *FRZB* and *WIF1* in canonical Wnt signaling pathway, and *ITGB3* and *NFATC1* in OCD signaling pathway (Table 1 and Fig. 3c). Spatial expressions of three genes with the highest fold-changes (one from each pathway: *WIF1*, *FGF13* and *ITGB3*) were assayed by in situ hybridization on sections of upper beaks at stages HH28/29. In situ hybridization supported transcriptome and RT-qPCR results that Ground Tits expressed higher level of *FGF13* but lower levels of *WIF1* and *ITGB3* than Great Tits (Fig. 3d). Their expressions were localized to the upper beak processes in both species.

### Gain of FGF13 and ITGB3 proteins affected beak morphology in chicken embryos

**Fig. 4:**
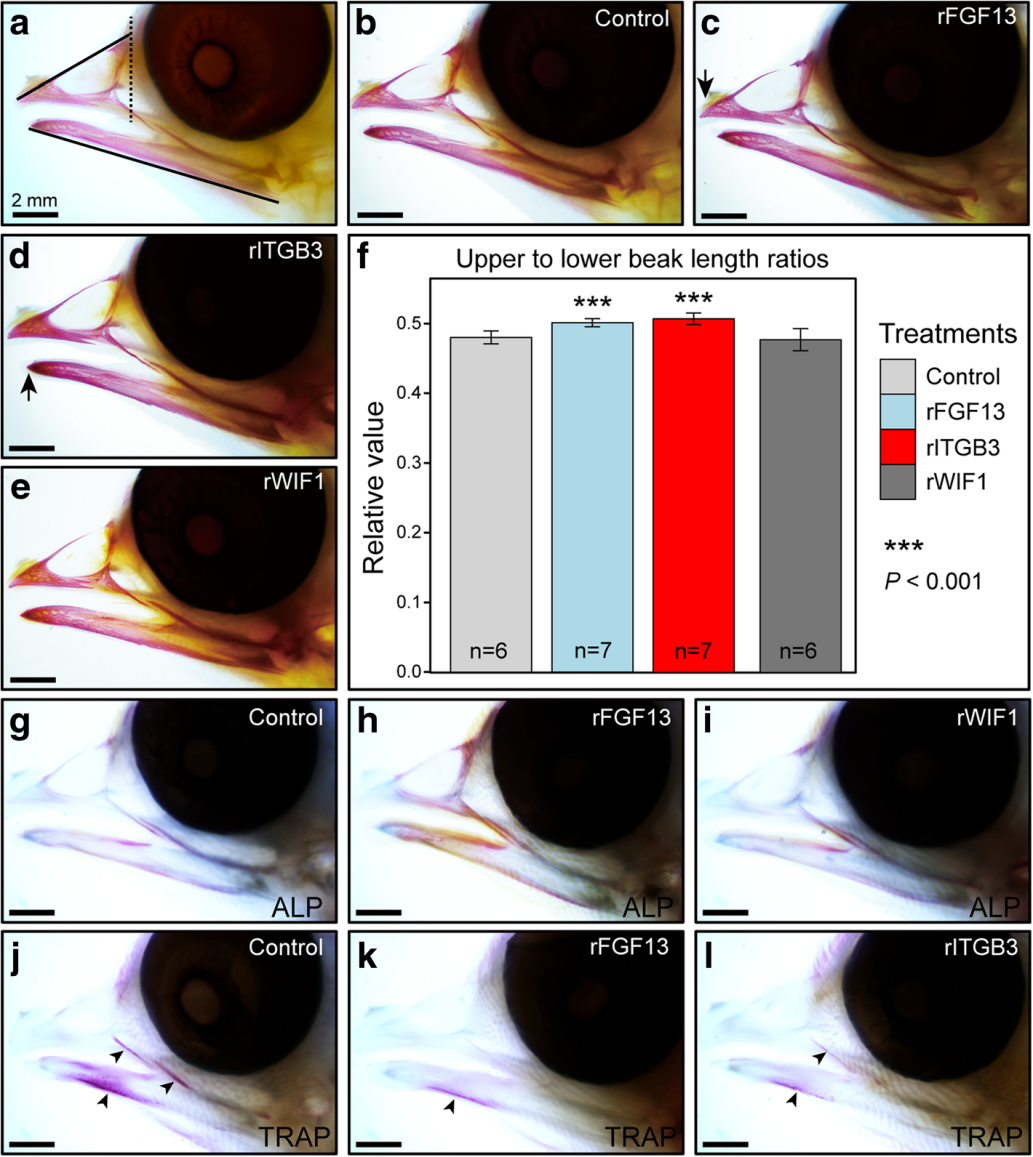
Injections of recombinant proteins affected beak development in chicken embryos. All embryos were injected at HH30 and were collected at HH38. HH, Hamburger and Hamilton stage. Bones of embryonic heads at HH38 were stained by alizarin red (**a-e**). **a.** The rule of measurements for the length of the upper and lower beak (arrow). **b.** Control embryos were treated with BSA. **c.** rFGF13-treated embryos had longer beaks. **d.** rITGB3 shortened the beak, especially the lower beak (arrow). **e.** No differences were observed in rWIF1-treated embryos. **f.** Quantification of treatment-dependent changes in length relative to control embryos. **g.** Whole mount ALP staining in the chicken head detected osteoblasts (stained purple) around the upper and lower beak skeleton (control). **h.** More robust ALP-positive staining in rFGF13-treated embryos (*n* = 3) demonstrated higher density of osteoblasts than control (*n* = 4). **i.** Levels of osteoblasts in rWIF1-treated embryos (*n* = 2) did not change. **j.** Whole mount TRAP staining detected more osteoclasts (stained purple) in the lower beak than the upper (arrowhead). **k-l.** Less TRAP-positive staining in rFGF13-treated (*n* = 2) and rITGB3-treated embryos (*n* = 3) demonstrated lower density of osteoclasts compared to control (*n* = 3)

Chicken embryos treated with recombinant FGF13 protein (rFGF13) and recombinant ITGB3 protein (rITGB3) showed an elevated proportion between upper and lower beak length relative to control embryos (Fig. 4a-d). These increases in ratios were statistically significant (Fig. 4f). However, we did not find significant decreases in ratios of upper to lower beak length in rWIF1-treated embryos compared to controls (Fig. 4e, f). Consistently, we also observed apparently increased amounts of osteoblasts in rFGF13-treated embryos but not in rWIF1-treated ones (Fig. 4g-i), and obvious reductions in amounts of osteoclasts in both rFGF13- and rITGB3-treated embryos (Fig. 4j-l).

## Discussion

### Evolutionary dynamics of the specialized beak in the Ground Tit

Geometric analysis separated more species pairs and detected differences among all species (see Additional file 1: Tables S4 and S5), while linear analysis only distinguished the Ground Tit from other parids (see Additional file 1: Tables S3), showing that the geometric analysis may be better to discriminate interspecific variations of parids than the linear analysis. Both morphometric analyses, however, could clearly separate the Ground Tit from all other parids mainly based on length and curvature variables. Although the curvature and length of the beak had no significant allometry, nearly half variation in curvature could be predicted by the variation of length, suggesting that beak curvature was co-evolved with beak length to some extent rather than completely independent. Ancestral reconstruction estimated that the long and decurved beak of the Ground Tit evolved from a short and straight ancestral type that is retained in most other extant parids. This morphological change in Ground Tits is likely to be an adaptation to a ground-living lifestyle in alpine steppe and meadow habitats created by the uplift of the Qinghai-Tibet Plateau [26].

Variation in beak morphology coincides with variation in diet and feeding behaviors in birds [1]. Ground Tits feed mostly on soil arthropods, such as insects and larvae, and occasionally on grass seeds and roots [22]. In sparse plateau environments with limited food resources [26], it is important for Ground Tits to be able to probe the turf and soil for food. A long and decurved beak is better suited for this terrestrial feeding behavior compared to the short and straight beaks of other parids, which occupy on arboreal niches.

The evolution of a long and decurved beak is also beneficial to Ground Tits for the construction of terrestrial nests in an open environment. Ground Tits nest in excavated burrows in ditches, pits, vertical banks or slopes [21]. Based on our field observations, Ground Tits excavate nests in banks or slopes by first using their beak and then using their talons to remove the soil. This burrowing behavior of Ground Tits probably also acted as a selective force in the development of their long and decurved beak.

### Changes in gene expression regulating beak development

Increasing researches have demonstrated that regulation of gene expression rather than functional coding change drives adaptively phenotypic evolution [5, 27], including some studies on evolution of beak morphology in birds [6–10]. Our results also showed that genes related to beak formation and development were not under selective constraints in terms of functional coding differences, but some of them were significantly differential expressed in embryonic beaks between Ground Tits and Great Tits, suggesting a transcriptional regulation in beak development. However, several other known genes, which have been discovered in contribution to beak morphogenesis of other birds [6–9, 28–30], were not found to be expressed differently between Ground Tits and Great Tits (see Additional file 1: Table S13). This inconsistency reflects that molecular programs of beak patterning are highly species-specific, which is a complex but flexible process involving multiple genes, signaling pathways, and reciprocal signaling interactions. Applications of different analyzing programs might also introduce the discordance by their own data-processing and statistical methods [31]. For example, DESeq and edgeR used in the current study found differential expression of *RALDH2* but NOISeq did not. In this case, DESeq and edgeR identified over 2000 differentially expressed genes that were not identified by NOISeq, which may result from poor FDR control for small sample sizes [31]. To improve true positives, we combined three approaches and conducted further validation.

RT-qPCR validation confirmed expression levels and patterns of five candidates, two of which, FGF13 and ITGB3, were functionally proved in chicken embryos. FGF13 is a member of the FGF signaling family (FGFs) in the FGF pathway. Previous reports have highlighted roles of FGFs in inducing neural crest cells to promote skeletal outgrowth of the beak [28, 29, 32, 33]. In our results, the injection of rFGF13 elevated the proportion between upper and lower beak length of chicken embryos and increased amounts of osteoblasts relative to control. We assumed that FGF13 might elongate the upper beak of embryos by increasing osteoblast formation, because FGFs have been involved in the generation of osteoblasts [34] that regulate bone deposition in the face and beak during embryonic development [35, 36]. However, we possibly underestimated the actual elongation of the beak by simply comparing ratios of upper to lower beaks, because rFGF13 increased osteoblasts in both upper and lower beaks of chicken embryos (Fig. 4h).

ITGB3, an adhesion receptor highly expressed in osteoclasts, is required for the interaction of osteoclasts with bone substrates during bone resorption [37, 38]. ITGB3-blocked mice have been found to stack more osteoclasts that are unable to adhere to bone substrates, and thus display a defect in bone resorption [37, 39]. Similarly, our functional experiments showed that rITGB3-treated chicken embryos held less osteoclasts relative to controls. We suggested that increases of ITGB3 protein probably boosted the adhesion of osteoclasts to bones, which drove embryos to consume more osteoclasts to participate in bone resorption. Osteoclast-dominated bone resorption has been discovered to negatively affect beak length during development [36]. Consequently, elevated proportions between upper and lower beak in rITGB3-treated embryos could presumably be results of the shortening of lower beaks, because more osteoclasts in lower beaks of chicken embryos could be used to take part in bone resorption than that in upper beaks (Fig. 4j). Moreover, we also observed reduced osteoclasts in rFGF13-treated embryos, reinforcing the negative regulation of osteoblasts to osteoclasts [40].

Taken together, our functional experiments preliminarily demonstrated the roles of the candidate genes, identified by RNA-Seq, in beak morphogenesis; *FGF13* and *ITGB3* might affect the beak morphology by regulating levels of osteoblasts and osteoclasts, respectively. Although *FGF13* and *ITGB3* were found to affect both upper and lower beak parts in chicken embryos, we infer that they could affect the upper beak more than the lower beak in embryos of Ground Tits and Great Tits according to their higher expression in upper beaks (Fig. 3d). In the context of the Ground Tit, therefore, we suggest that a long decurved upper beak could be most likely regulated by *FGF13*- and *ITGB3*-mediated osteoblasts and osteoclasts.

## Conclusion

Our results show that the ground-oriented Ground Tit evolved a comparatively longer and more decurved beak from a short straight form which is similar to most other Paridae species. The adaptive variation of the beak morphology is likely associated with changes in gene expression rather than mutations in coding sequences. Our functional assays also show that changing transcriptional activities of genes may affect beak morphology by modulating osteoblasts and osteoclasts.

## Methods

### Morphometric analyses

We examined 349 study skin specimens from 13 Paridae species with body mass information recorded in the National Zoological Museum of the Institute of Zoology, Chinese Academy of Sciences. We used a digital caliper to measure the length (from the rostral edge of the nares to the tip), depth (at the nares) and width (at the nares) of the upper beak (Fig. 1a) that determines the species-specific morphology of the avian beak [41] and reflects the functional biomechanical properties of the entire beak [42, 43]. Beak length data of 282 specimens were from our previous research [23]. All measurements were standardized by the cube root of body mass to account for body size dependence [44] (see Additional file 1: Table S14; see Additional file 2). To test phylogenetic independence for linear measurements (length, depth and width), we used Blomberg’s K [45] and Pagel’s λ [46] to assess their phylogenetic signals under a Brownian motion (BM) model based on a published phylogenetic tree of parids [47]. For both indices, a value close to zero indicates phylogenetic independence and a value of one indicates that traits are evolving under BM. Subsequently, we evaluated the overall variation in linear measurements using Hotelling-Lawley’s test and conducted LDAs to discriminate Ground Tits from other parids.

We also compared the shape of the upper beak in the Ground Tit with that in other parids using geometric morphometric analysis. A total of 352 lateral images of upper beaks of the skin specimens were used in the geometric analysis (see Additional file 3), including 292 images collected from our recently published work [23] and 60 newly photographed images using a microscopic image collecting system. We placed 3 landmarks and 116 sliding semi-landmarks on each beak image (Fig. 1a) to characterize the shape of the upper beak using TPSDIG [48]. To eliminate the effect of location, direction and scale on beak shape, we used generalized least square superimposition to rotate, translate and scale landmark coordinates in TPSRELW [49]. We firstly tested phylogenetic signals on both Procrustes coordinate and centroid size with 10,000 random permutations [50] using *Collect Statistics on Tree Set* option in Morpho J [51]. To assess allometric relationship between beak shape and size, additionally, we performed a multivariate regression of Procrustes coordinates onto centroid size under *Regression* option [52]. Procrustes ANOVA and CVA were used to analyze interspecific variations of the beak shape [51]. The CVA is a method used to extract the axes with the greatest interspecific differences and generate a matrix of pairwise Mahalanobis distances [53]. DFA was performed to examine the separation between each species pair [54]. All specimens used are adults without significant variations in beak size and beak shape between males and females (see Additional file 1: Table S15). Non-parametric Wilcoxon rank sum test was used to examine the difference between sexes.

### Ancestral state reconstruction

To estimate ancestral beak morphology of the Ground Tit and other parids, we performed an ancestral state reconstruction for beak morphology based on the phylogenetic tree of parids, including beak size and beak shape. The ancestral states of the beak size were inferred using a maximum likelihood method implemented in R using the APE package [55], while those of the beak shape were estimated based on Procrustes coordinates with *map onto phylogeny* option using squared-change parsimony method [56] in Morpho J.

### Orthology and evolutionary analyses

Genomic coding sequences from five avian species, including the Ground Tit [19], Great Tit (NCBI, Parus_major1.1), Medium Ground Finch (NCBI, GeoFor_1.0), Atlantic Canary (NCBI, SCA1) and American Crow (NCBI, ASM69197v1), were used in this analysis. The longest transcript isoform was retained for each gene, and genes shorter than 150 bp were discarded. Putative orthologous among above five species were identified using an all-against-all BLAST with an E value 1E-10. Each orthologues gene was aligned and trimmed. Phylogeny and divergence time were estimated using a substitution rate of ~3.3 × 10^−3^ substitutions per site per million years [57] in BEAST 2 [58]. Based on this phylogeny, we estimated lineage-specific evolutionary information, such as dN, dS and dN/dS, using Codeml program in PAML package under the free-ratio model [59]. To reduce errors and avoid saturated mutations, we retained only the genes with N*dN > 1, S*dS > 1 and dS < 1 [60]. The likelihood values between the alternative and null models were compared to test differences in dN/dS ratios among lineages [61]. *P*-values were adjusted for multiple tests with false discovery rate (FDR) [62]. FDR-adjusted *P*-values below 0.05 were used as the threshold for statistical significance. Wilcoxon rank sum tests were used to test whether the mean dN/dS value of the Ground Tit differs significantly from those of other lineages.

### Selection and identification of developmental stages

At early stages of embryonic development, initiation, migration and differentiation of neural crest cells contribute to avian beak morphogenesis [63, 64]. Beak morphogenesis involves multiple facial prominences, including FNM, lateral nasal prominence (LNP), maxillary prominence (MXP) and mandibular prominence (MDP) [65]. The FNM, LNP and MXP fuse the upper beak. The internal bony scaffolds of the upper beak are composed of the pnc and pmx, which are formed from the differentiation of mesenchyme cells derived from neural crest cells [64, 66]. The pmx is the most prominent functional and structural component of the upper beak, and its variations are often correlated with beak diversity [67]. During Hamburger and Hamilton (HH) [68] stages 28-30, the pmx forms and shapes while the pnc cease its expansion [9]. All embryos used in this study, thus, were matched with HH28 or HH29. We examined embryonic stages of the two tits based on the staging series of chicken, quail and some finches. Given that heterochrony of embryonic development exists between precocies and altrices [69], we referenced altricial embryonic days (ca. E6-6.5) corresponding to HH28/29 (see Additional file 1: Table S16).

### Embryo collection and tissue preparation

We collected embryos during two breeding seasons (April 2013 to July 2013 and April 2014 to July 2014) in Nagqu, Tibet (Ground Tit) and Zuojia, Jilin (Great Tit) (see Additional file 1: Figure S4). In the breeding season, we observed and traced breeding pairs to locate nests and confirmed laying and hatching date according to their reproductive behaviors. Ground Tit females lay clutches of 4-9 eggs, and Great Tits lay 5-12 eggs, one per day [20]. Both species start their incubation upon laying their last eggs. We determined the initiation of the incubation according to behaviors of breeding pairs, such as feeding by males, staying time of females in nests. When these behaviors were observed, we dug nest burrows (Ground Tits) and examined nest boxes (Great Tits) to collect eggs and then incubated them in a micro-incubator at 38 °C. At E6 and E6.5, we opened a few eggs to determine desired stages under stereoscope according to external identification features (see Additional file 1: Table S16); HH28 of Ground Tits matched with E6.5 and that of Great Tits with E6. We collected a total of 11 stage-matched Ground Tit embryos and 12 stage-matched Great Tit embryos during two breeding seasons (see Additional file 1: Figure S5). Embryos were cleaned with cold 1× PBS and then stored in RNA*hold* (TranGen) at 4 °C. Eight stage-matched Ground Tit and 9 Great Tit embryos were dissected for RNA extraction. Three matched embryos of each species were fixed in 4% paraformaldehyde (PFA) solution for in situ hybridization. The field collection of embryos was performed with the permission of the State Forestry Administration of China and conformed to the National Wildlife Conservation Law of China.

### Transcriptome sequencing, quality control and reads mapping

Total RNA was extracted from four embryonic upper beaks from each species using TRIzol reagent (Invitrogen). The quality of extracted RNA samples was examined using a Nanodrop spectrophotometer and an Agilent 2100 Bioanalzyer. Sequencing libraries (non-strand-specific) were constructed for two qualified RNA samples from each species according to the manufacturer’s protocol (Illumina). RNA sequencing was performed based on 100 bp paired-end reads using an Illumina HiSeq 2000 in the *Novogene Bioinformatics Institute* (Beijing, China), which produced a total of 21.91 GB raw reads. We used Cutadapt to remove adapters and reads with >5% unidentified nucleotides [70], used FASTX-Toolkit (http://hannonlab.cshl.edu/fastx_toolkit) to remove reads with length of <90 and reads with <90% bases that had Phred quality score > 20, as well as used Trimmomatic to trim and pair reads using default setting [71]. The resulting 19.61 GB clean reads were retained for subsequent analyses: 21,689,370 and 22,209,206 reads for two Ground Tits and 28,570,866 and 25,599,330 reads for two Great Tits. An assessment for clean reads showed high quality with Q20 > 99.28% and Q30 > 95.55% (see Additional file 1: Table S17). The high-quality reads of Ground Tits and Great Tits were mapped to respective genomes by TopHat 2.09 with default parameters [72].

### Gene quantification and differential expression analysis

We quantified gene expression based on two data types; the simple read counts calculated by HTSeq-count [73], and the transformed TPM (transcripts per million) computed using a simple formula [74] from the measured FPKM (fragments per kilobase of exon model per million mapped reads) by Cufflinks [75]. The application of TPM was to take into account differences of library sizes between samples and the different length of genes between species. We identified 12,232 orthologous genes between the Ground Tit and the Great Tit. Genes were considered to be confidently expressed when their read counts ≥10. But the genes with more than 2-folds differences within species were removed. Final 9217 genes were retained for subsequent differential expression analysis. We compared gene expression patterns between Ground Tits and Great Tits using three different R programs, DESeq, edgeR and NOISeq. DESeq and edgeR were employed to input raw read counts, normalize counts with library size using their own methods, and perform differential expression analysis based on negative binomial distribution [76, 77]. Non-parametric NOISeq was applied to compare the TPM based on noise distribution that considers differences within species [78]. Genes were considered to be differentially expressed if they were observed to have a 2-folds change in expression and a FDR-adjusted *P*-value less than 0.001 [79]. To decrease false positives, we considered only genes that were identified by all three programs to have significantly differential expression with the same pattern (up-regulation or down-regulation). We used KOBAS [80] to enrich KEGG pathways and GO categories for these identified genes, with a focus on the pathways associated with the formation of cartilage and bone [35, 81].

### RT-qPCR and in situ hybridization

To verify expression levels and patterns of genes identified by transcriptome analyses, we performed RT-qPCR for eight candidates in upper beaks of embryos because of limitation of embryo numbers (four replicates per species). Seven of them were selected from pathways related to bone development with the highest *P*-values and fold changes (Table 1). *FRZB*, although was not the one with the highest *P*-value and fold change, was annotated with a function in regulation of cartilage development (see Additional file 1: Table S12), therefore we also considered this gene for our following RT-qPCR. All primers were designed based on identical sequences of the relevant genes in both species (see Additional file 1: Table S18). Four isolated RNA samples from each species were converted to cDNA in a 50 μl reaction using *TransScript* SuperMix (TranGen). cDNA, primers and *TransStart* SuperMix (TranGen) were mixed in a 20 μl reaction to amplify each gene. RT-qPCR was run in triplicate on a Roche LightCycler 96 using a program: 94 °C for 30 s, 40 cycles at 94 °C for 5 s, 60 °C for 15 s and 72 °C for 10 s. The relative expression (∆Cq) of all genes was normalized to glyceraldehyde-3-phosphate dehydrogenase (*GAPDH*), which is inversely proportional to the real expression levels. We examined significance in relative expression using Student’s t-test. Expression differences were calculated using the -∆∆Cq method [82]. In situ hybridization was performed as previously described [83] on paraffin sections of embryonic heads at stage HH28/29 to assay spatial patterns of gene expression. Sections were hybridized with fluorescein-labeled RNA probes to *FGF13*, *WIF1* and *ITGB3* and hybridization signals were visualized using confocal epifluorescence.

### Functional test in chicken embryos

Chicken embryos were manipulated using a biochemical strategy to test if changes in expression of the candidate genes, *WIF1*, *FGF13* and *ITGB3*, can affect beak morphological traits. Eggs were incubated at 38 °C and 60-70% humidity. At HH30, 10 μl of recombinant mouse ITGB3 protein (200 ng/μl) (R&D Systems) (*n* = 15), recombinant mouse WIF1 protein (250 ng/μl) (R&D Systems) (n = 15), and FGF13 recombinant protein (50 ng/μl) (Novus Biologicals) (n = 15), was injected into the vitelline vein using a sterilized glass needle. Prior to injection, we opened an ~2 cm oval window in minor diameter using a scalpel and removed the external membrane using fine forceps. Control embryos (n = 15) were treated with 0.1% bovine serum albumin (BSA). Surviving embryos were collected at HH38, fixed with 4% PFA and stored in 100% methanol after gradient dehydration. We collected a total of 43 survived embryos consisting of 13 controls, 12 rFGF13 treatments, 10 rITGB3 treatments, and 8 rWIF1 treatments.

Embryos were stained with 0.1% Alizarin Red, and cleared in gradient glycerol [84]. Lateral images of the embryonic specimens were captured on a stereo microscope. We defined upper and lower beak measurements in ImageJ (NIH) following Ealba’s method [36]: upper beak measurement was taken from the tip of the nasal bone in the center of the maxilla to the distal tip of the premaxilla, and lower beak measurement was made from the proximal tip of the angular bone to the distal tip of the dentary bone (Fig. 4a). The values were presented as ratios of upper to lower beak measurements to eliminate individual and stage variations, and they were compared between treatments and controls by Student’s t-test. To detect osteoblasts and osteoclasts in treatments separately, alkaline phosphatase (ALP) and tartrate-resistant acid phosphatase (TRAP) were stained in whole-mount embryos with Fast Red following Leukocyte Alkaline Phosphatase Kit and Acid Phosphatase Leukocyte kit (Sigma) protocols.

## Additional files

Additional file 1: Supplementary materials. Included are 8 supplementary figures (Figures S1-S8) and 18 supplementary tables (Tables S1-S18). (PDF 1215 kb)

Additional file 2: The beak size data of 13 parids used in this study. (XLS 93 kb)

Additional file 3: The beak shape data of 13 parids used in this study. (XLS 91 kb)

Additional file 4: The description of expression abundance of the 623 DEGs. (XLS 217 kb)

## Abbreviations

ALP: Alkaline phosphatase
BSA: Bovine serum albumin
CVA: Canonical variate analysis
FDR: False discovery rate
FNM: Frontal nasal mass
FPKM: Fragments per kilobase of exon model per million mapped reads
GO: Gene ontology
HH: Hamburger and Hamilton stage
KEGG: Kyoto encyclopedia of genes and genomes
LDA: Linear discriminant analysis
LNP: Lateral nasal prominence
MDP: Mandibular prominence
MXP: Maxillary prominence
PBS: Phosphate buffered saline
PFA: Paraformaldehyde
RT-qPCR: Reverse transcription quantitative PCR
TPM: Transcripts per million
TRAP: Tartrate-resistant acid phosphatase
