# Supplementary material for: Evolution of beak morphology in the Ground Tit revealed by comparative transcriptomics
Source: Front Zool. 2017 Dec 21;14:58. doi: 10.1186/s12983-017-0245-6 (PMC5740785; doi:10.1186/s12983-017-0245-6)
Supplement: Supplementary file 1 — Supplementary materials. Included are 8 supplementary figures (Figures S1-S8) and 18 supplementary tables (Tables S1-S18). (PDF 1215 kb) [file 12983_2017_245_MOESM1_ESM.pdf]

Title: Evolution of beak morphology in the Ground Tit revealed by comparative transcriptomics

Yalin Cheng[1, 4, †], Bin Gao[1, †], Haitao Wang[2, †], Naijian Han[1], Shimiao Shao[1, 4], Shaoyuan Wu[3], Gang Song[1], Yong E. Zhang[1], Xiaojia Zhu[1, 4], Xin Lu[5], Yanhua Qu[1, *] and Fumin Lei[1, 4, *]

Address: [1]Key Laboratory of Zoological Systematics and Evolution, Institute of Zoology, Chinese Academy of Sciences, Beijing 100101, China; [2]School of Life Sciences, Northeast Normal University, Changchun 130024, China; [3]School of Life Sciences, Jiangsu Normal University, Xuzhou 221116, China; [4]University of Chinese Academy of Sciences, Beijing 100049, China; [5]Department of Ecology, College of Life Sciences, Institute for Advanced Studies, Wuhan University, Wuhan 430072, China

E-mail: Yalin Cheng, chengyalin@ioz.ac.cn; Bin Gao, rahello1234@163.com; Haitao Wang, wanght402@nenu.edu.cn; Naijian Han, hannaijian@163.com; Shimiao Shao, 767843291@qq.com; Shaoyuan Wu, shaoyuanwu@outlook.com; Gang Song, songgang@ioz.ac.cn; Yong E. Zhang, zhangyong@ioz.ac.cn; Xiaojia Zhu, zhuxiaojia@ioz.ac.cn; Xin Lu, luxinwh@163.com; Yanhua Qu, quyh@ioz.ac.cn; Fumin Lei, leifm@ioz.ac.cn

[†] These authors contributed equally to this work

[*] Corresponding authors

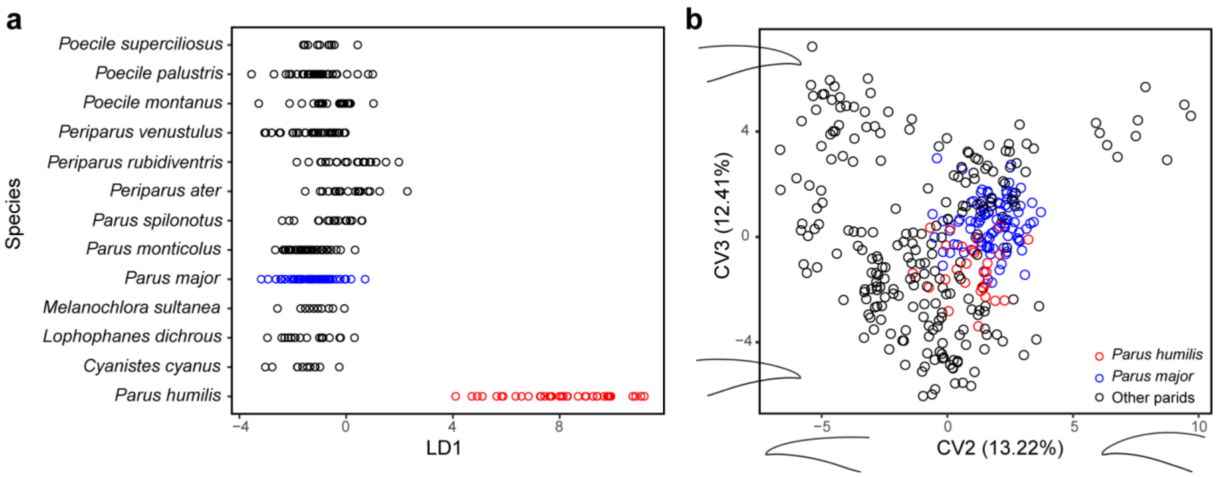

Figure S1. LDA with beak length and CVA with beak shape. **a.** LDA with only beak length showed that Ground Tits are clearly separated from other parids. **b.** CV2 and CV3 cannot discriminate Ground Tits from other parids. CV2 predicted the variations between the Sultan Tit and other parids. The beak shape profiles were generated by Morpho J, which indicate the changes in beak shape along the axes.

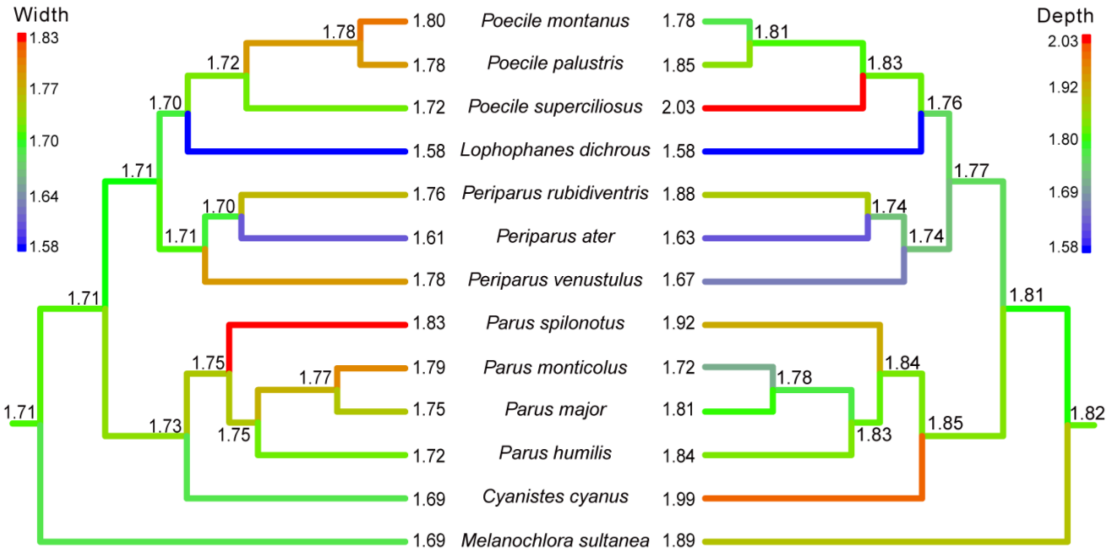

Figure S2. Reconstruction of the ancestral state for beak width and depth. The node labels indicate the width and depth of ancestral beaks. From blue to red, the greater the value and the wider or deeper the beak.

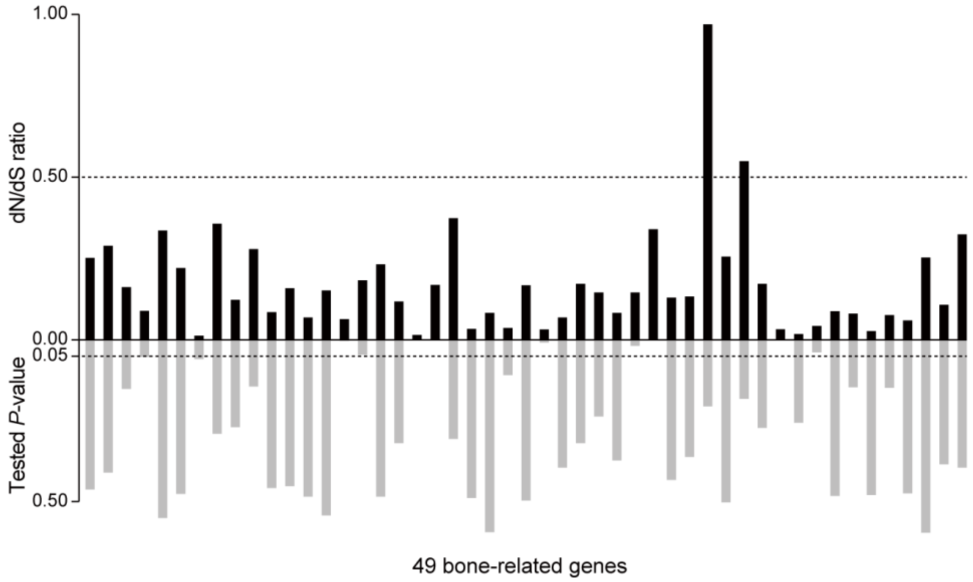

Figure S3. The corresponding dN/dS ratios and likelihood-ratio-tested values for the 49 bone development related genes. None of the 49 bone-related genes was assigned both a dN/dS > 0.5 and a *P*-value < 0.05.

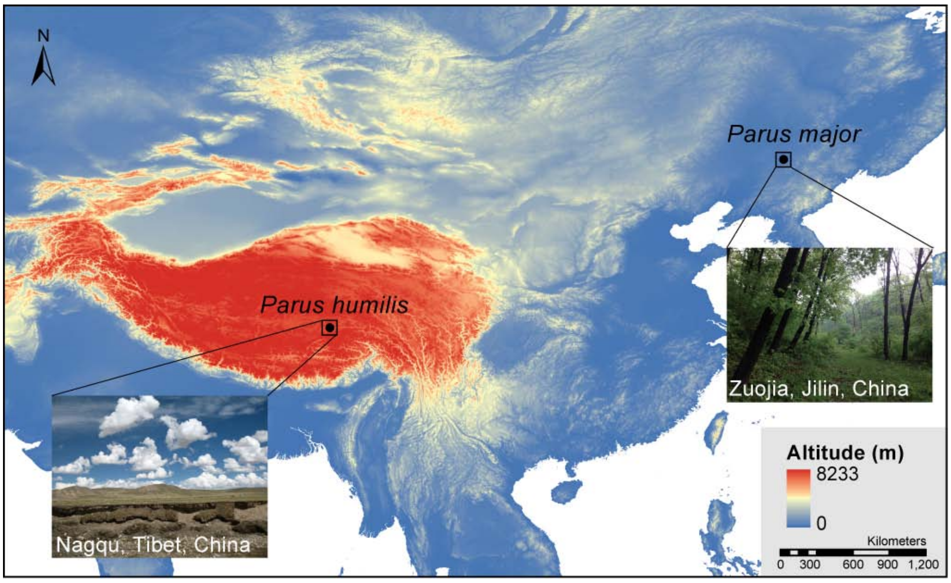

Figure S4. Sampling sites for Ground Tits and Great Tits embryos in China. The map was drawn using ArcGIS 9.3 (ESRI, http://www.esri.com/) [1] based on the global 30 arc-seconds digital elevation data (https://lta.cr.usgs.gov/GTOPO30) that is available from the U.S. Geological Survey (USGS). The habitat photographs embedded were taken by Yalin Cheng during embryos sampling in the field (Nagqu and Zuojia).

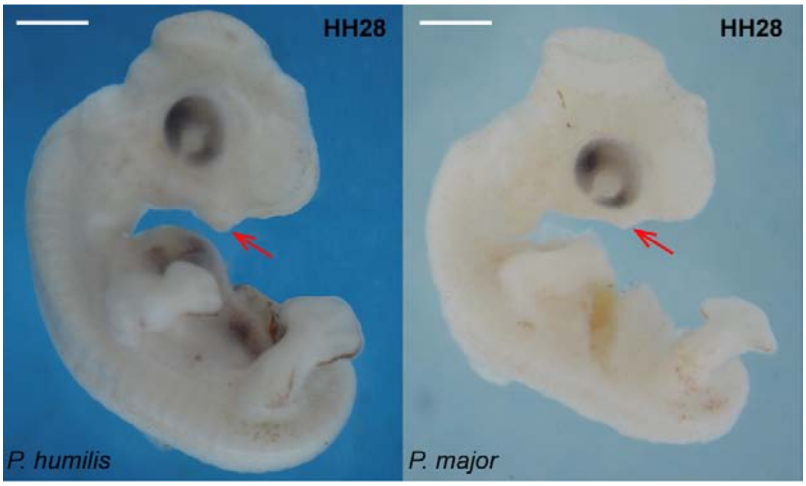

Figure S5. The embryos of the Ground Tit and the Great Tit at stage 28. At stage 28, beak outgrowth and the grooves between the digits or toes are distinct, and the egg tooth is not present. The red arrows indicate the upper-beak. Scale bar are equal to1 mm. HH, Hamburger and Hamilton stage.

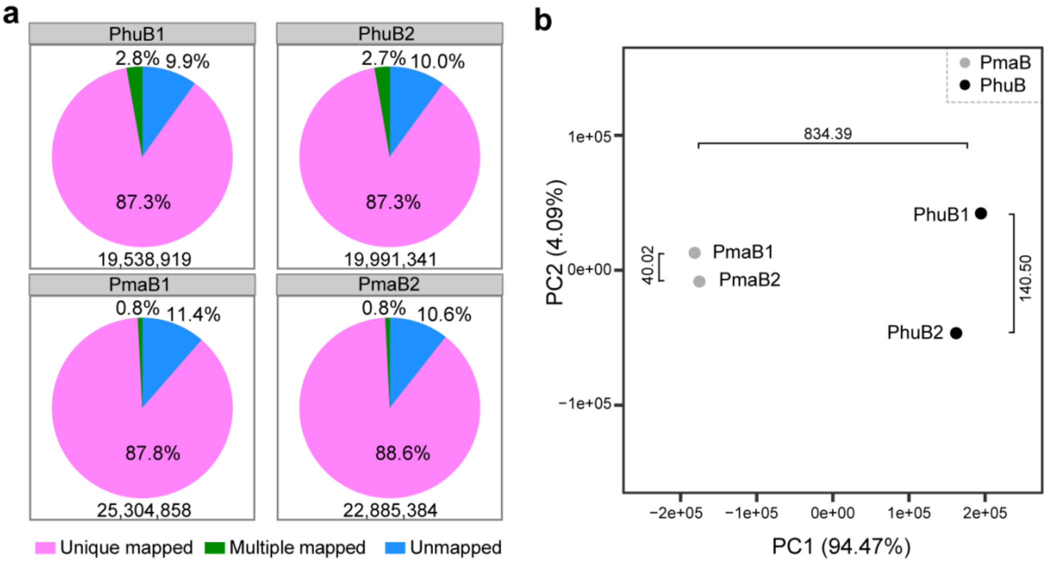

Figure S6. Statistic for reads mapping and analysis for expression. **a.** The results of reads mapping for each sample. Ground Tits and Great Tits have the same mapping rate. Numbers under the pies are the mapped reads. **b.** PCA results for the expression of overall gene set show higher variation between species than within species. Numbers above or beside lines are mean discrepancy of read counts between or within species. PhuB1, replicate 1 of Ground Tit beak; PhuB2, replicate 2 of Ground Tit beak; PmaB1, replicate 1 of Great Tit beak; PmaB2, replicate 2 of Great Tit beak.

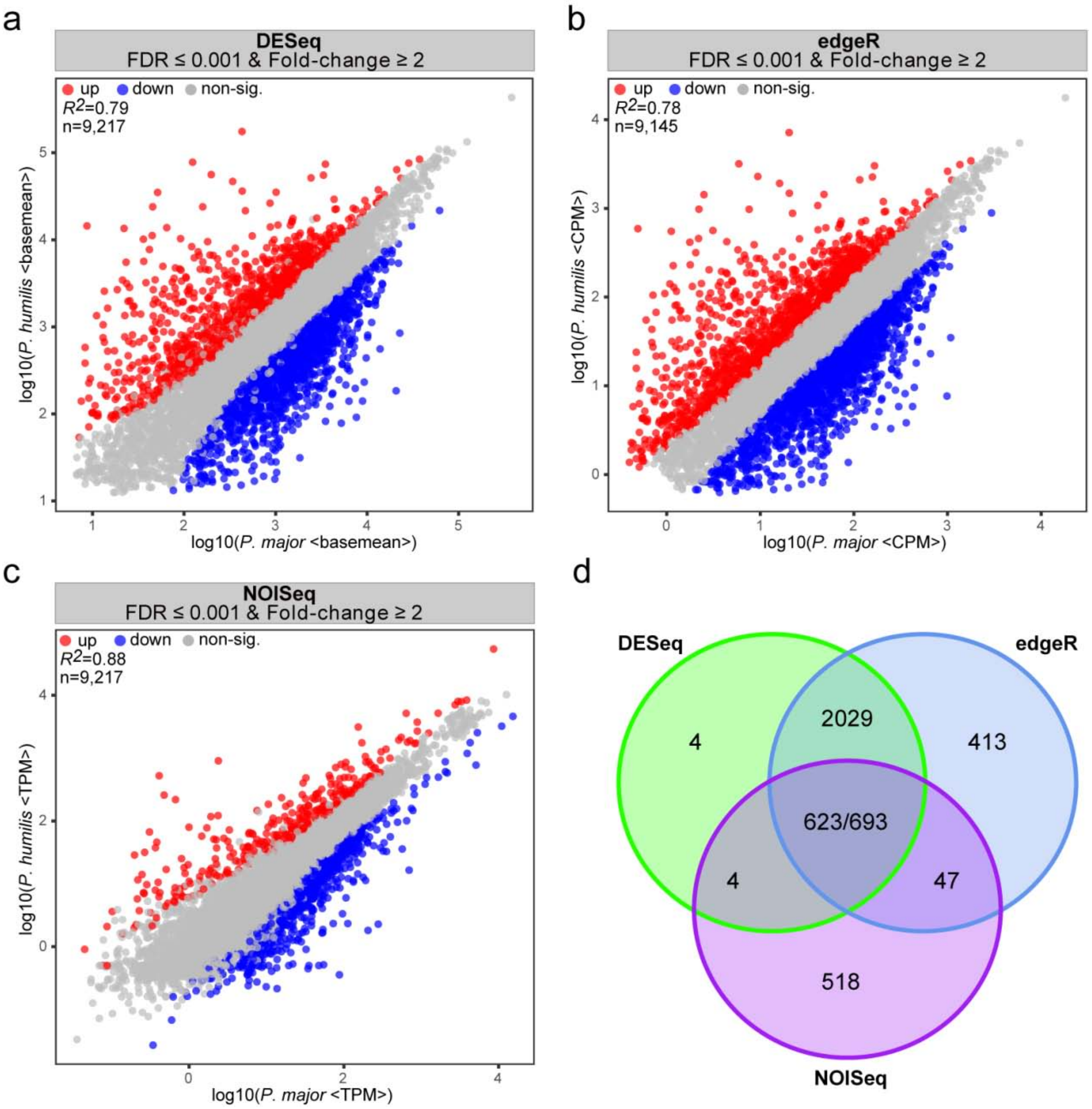

Figure S7. Pairwise comparisons of gene expression between Ground Tits and Great Tits. **a.** Gene expression qualified by DESeq. **b.** Gene expression qualified by edgeR. **c.** Gene expression qualified by NOISeq. The red, blue and gray colors (**a-c**) represent the up-regulated, down-regulated and non-significantly changed genes, respectively. R square showed regression coefficients between the expressions of Ground Tits and Great Tits. CPM is the count per million. TPM is the transcript per million. **d.** The Venn diagram shows the differentially expressed genes that were identified using DESeq, edgeR and NOISeq. The number on the left of the backslash (**d**) is the finally differentially expressed genes that equip significant difference and the same expression patterns in three approaches. The number on the right of the backslash (**d**) is the only genes with significant difference in three approaches. Different colors represent different analyses approaches.

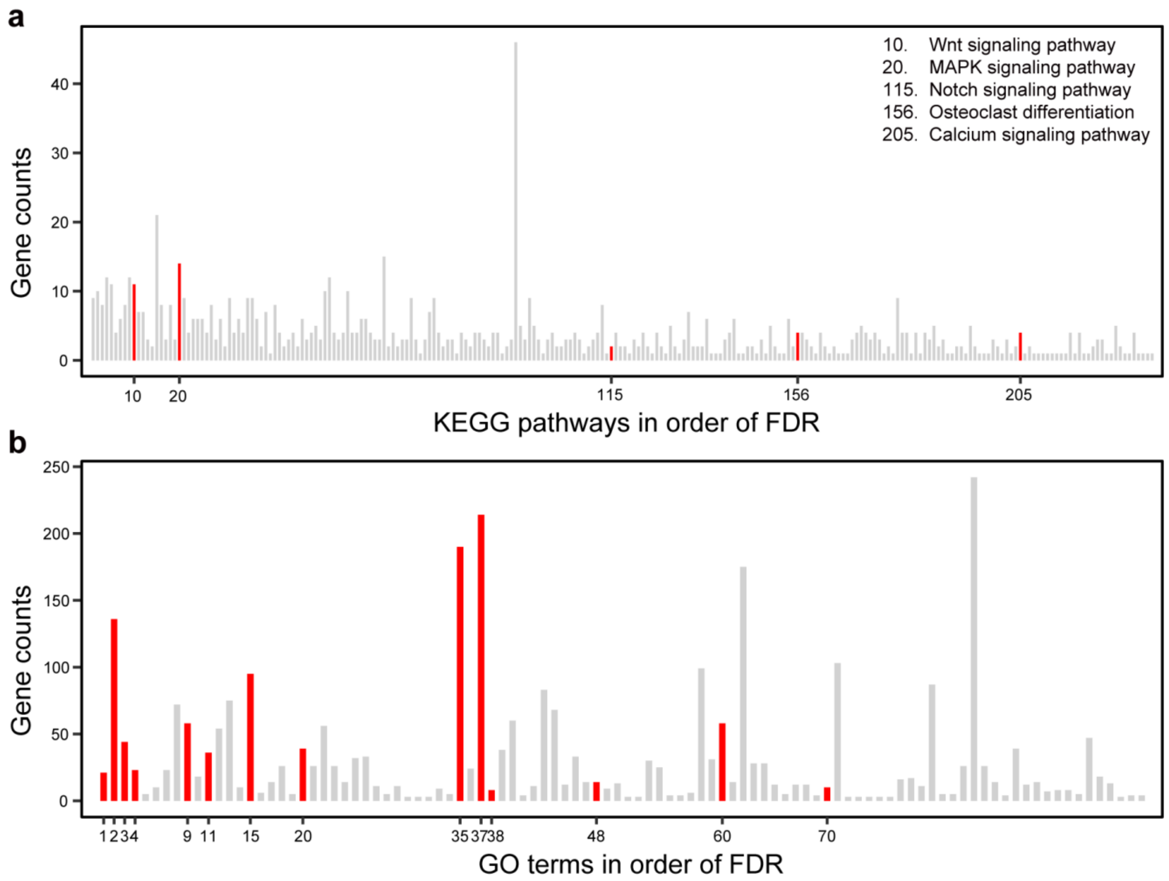

Figure S8. KEGG and GO enrichment for differentially expressed genes. **a.** KEGG pathway enrichment. Red bars indicate the five pathways that are probably associated with beak morphogenesis. **b.** Top 100 enriched GO terms. The red bars represent function categories that are probably associated with beak development and morphogenesis, which are mainly clustered into Top 20 terms. 1, cartilage development; 2, anatomical structure morphogenesis; 3, tissue morphogenesis; 4, connective tissue development; 9, organ morphogenesis; 11, skeletal system development; 15, tissue development; 20, embryonic morphogenesis; 35, system development; 37, multicellular organismal development; 38, regulation of cartilage development; 48, appendage morphogenesis; 60, anatomical structure formation involved in morphogenesis; 70, chondrocyte differentiation.

Table S1. Phylogenetic signals were tested for beak size.

Linear measurements	Blomberg's K [2]		Pagel's λ [3]	
	K	***P***	***λ***	***P***
Length	0.979	0.163	6.610696e-05	1
Width	0.695	0.371	6.610696e-05	1
Depth	0.637	0.463	6.610696e-05	1

Two methods to test if the beak length, width and depth evolved with background phylogenetic signals. *K* & *λ* are indices of phylogenetic signals from two different methods respectively. A value close to zero indicates phylogenetic independence and a value of one indicates that traits are evolving under BM. *P* is the value of statistical significance.

Table S2. Eigenvalues of discriminant functions.

	LD1	LD2	LD3
% variance	92.99	5.04	1.97
Eigenvalue	367.849	19.942	7.781
Length	-4.448	-0.142	0.203
Width	4.682	-3.297	9.214
Depth	2.261	8.489	-0.893
% variance	70.48	29.52	
Eigenvalue	20.320	8.511	
Width	3.848	9.256	
Depth	-8.439	-0.413	

Above the dashed line is the LDA based on beak length, width and depth. Below the dashed line is the LDA based on beak width and depth.

Table S3. Percentages of species classification obtained from linear discriminant analyses (LDA) based on three different datasets; LDA with length, width and depth (upper panel), LDA without length (middle panel), and LDA with only length (lower panel).

Species	Numbers	Phu	Ccy	Ldi	Msu	Pma	Pmo	Psp	Pat	Pru	Pve	Pmon	Ppa	Psu
**Phu**	39	**100**												
**Ccy**	11		**36.36**		10.00								2.50	8.33
**Ldi**	16			**56.25**			2.56		11.11	9.09	11.43	4.55	2.50	
**Msu**	10				**10.00**									
**Pma**	68		54.55	18.75	80.00	**85.29**	43.59	58.82	11.11	22.73	34.29	59.09	62.50	41.67
**Pmo**	39					4.41	**35.90**	5.88			14.29	4.55	7.50	
**Psp**	17							**5.88**		4.55				
**Pat**	18			18.75					**61.11**	4.55			2.50	
**Pru**	22					2.94		5.88	11.11	**50.00**		4.55	5.00	8.33
**Pve**	35			6.25		4.41	15.38		5.56	4.55	**34.29**	13.64	5.00	
**Pmon**	22						2.56	5.88				**9.09**	2.50	
**Ppa**	40					1.47		5.88			5.71		**2.50**	
**Psu**	12		9.09			1.47		11.76		4.55			7.50	**41.67**
**Phu**	39	**15.38**	18.18		10.00	4.41	2.56		11.11	18.18	2.86		7.50	25.00
**Ccy**	11	2.56	**27.27**		20.00			5.88					2.50	8.33
**Ldi**	16			**37.50**					27.78	4.55	17.14	4.55		
**Msu**	10				**0.00**									
**Pma**	68	71.79	45.45	37.50	60.00	**86.76**	38.46	58.82	11.11	50.00	31.43	63.64	65.00	25.00
**Pmo**	39	2.56				4.41	**30.77**	11.76		4.55	11.43	22.73	5.00	
**Psp**	17							**5.88**		9.09				8.33
**Pat**	18	2.56		18.75	10.00		2.56		**22.22**					
**Pru**	22									**0.00**				
**Pve**	35			6.25		1.47	25.64		22.22		**31.43**	9.09	7.50	
**Pmon**	22											**0.00**		
**Ppa**	40	5.13				1.47		5.88	5.56	9.09	5.71		**5.00**	
**Psu**	12		9.09			1.47		11.76		4.55			7.50	**33.33**
**Phu**	39	**100**												
**Ccy**	11		**0.00**											
**Ldi**	16			**0.00**										
**Msu**	10				**0.00**									
**Pma**	68		100	93.75	100	**97.05**	97.44	82.35	55.55	50.00	100	95.45	92.50	91.67
**Pmo**	39						**0.00**							
**Psp**	17							**0.00**						
**Pat**	18								**0.00**					
**Pru**	22			6.25		2.95	2.56	17.65	44.45	**50.00**		4.55	7.50	8.33
**Pve**	35										**0.00**			
**Pmon**	22											**0.00**		
**Ppa**	40												**0.00**	
**Psu**	12													**0.00**

Dash lines divided this table into three panels. *Parus humilis*, Phu; *Cyanistes cyanus*, Ccy; *Lophophanes dichrous*, Ldi; *Melanochlora sultanea*, Msu; *Parus major*, Pma; *Parus monticolus*, Pmo; *Parus spilonotus*,

Psp; *Periparus ater*, Pat; *Periparus rubidiventris*, Pru; *Periparus venustulus*, Pve; *Poecile montanus*, Pmon; *Poecile palustris*, Ppa; *Poecile superciliosus*, Psu. The bolded numbers stand for the percentages (%) of the correct classification. The misclassifications are not bolded.

Table S4. Percentages of species classification in discriminant function analysis (DFA) for beak shape.

Species	Numbers	Phu	Ccy	Ldi	Msu	Pma	Pmo	Psp	Pat	Pru	Pve	Pmon	Ppa	Psu
**Phu**	27	**100**												
**Ccy**	11		**81.82**											11.11
**Ldi**	16			**68.75**		2.13						4.35		
**Msu**	10				**90.00**									
**Pma**	94					**84.04**	10.00	5.56						
**Pmo**	30			12.50		7.45	**83.33**	11.11						
**Psp**	18				10.00	4.26	6.67	**83.33**						
**Pat**	21			6.25					**100**					
**Pru**	22			6.25						**95.45**		4.35		
**Pve**	33					1.06					**100**			
**Pmon**	23					1.06				4.55		**86.96**	5.26	
**Ppa**	38			6.25								4.35	**86.84**	
**Psu**	9		18.18										7.89	**88.89**

Parus humilis, Phu; *Cyanistes cyanus*, Ccy; *Lophophanes dichrous*, Ldi; *Melanochlora sultanea*, Msu; *Parus major*, Pma; *Parus monticolus*, Pmo; *Parus spilonotus*, Psp; *Periparus ater*, Pat; *Periparus rubidiventris*, Pru; *Periparus venustulus*, Pve; *Poecile montanus*, Pmon; *Poecile palustris*, Ppa; *Poecile superciliosus*, Psu. The percentages (%) of the correct classification are bolded.

Table S5. Pairwise Mahalanobis distances generated by canonical variate analysis (CVA). Permutation tests (10,000 rounds) showed significant differences in Mahalanobis distances among all species (*P* <0.0001).

Species	Numbers	Ccy	Ldi	Msu	Pma	Pmo	Psp	Pat	Pru	Pve	Pmon	Ppa	Psu
**Ccy**	11												
**Ldi**	16	12.0986											
**Msu**	10	15.3848	14.1106										
**Pma**	94	10.0040	7.4050	11.8536									
**Pmo**	30	11.2505	8.8318	12.1207	5.3949								
**Psp**	18	11.2959	9.2303	12.4521	6.3636	6.9061							
**Pat**	21	13.5773	9.4857	15.1331	9.6122	10.4167	10.6247						
**Pru**	22	11.126	8.7852	15.1745	7.6644	9.0773	9.7788	9.3509					
**Pve**	33	12.5319	10.0605	14.8975	8.3592	8.2671	9.0823	9.8552	10.1765				
**Pmon**	23	11.1275	8.2600	14.3342	7.1462	8.1532	8.5811	8.7652	8.5539	8.9510			
**Ppa**	38	10.1591	10.0052	14.5391	7.2533	8.8696	8.9495	10.988	8.9621	10.4841	7.1648		
**Psu**	9	12.1925	11.7335	15.6712	9.5344	10.8081	10.5718	13.462	11.0289	13.4569	10.9551	10.2453	
**Phu**	27	20.1646	15.1081	18.5278	15.7983	16.2214	17.0714	15.53	16.6548	16.8331	16.2586	17.4891	19.4309

Parus humilis, Phu; *Cyanistes cyanus*, Ccy; *Lophophanes dichrous*, Ldi; *Melanochlora sultanea*, Msu; *Parus major*, Pma; *Parus monticolus*, Pmo; *Parus spilonotus*, Psp; *Periparus ater*, Pat; *Periparus rubidiventris*, Pru; *Periparus venustulus*, Pve; *Poecile montanus*, Pmon; *Poecile palustris*, Ppa; *Poecile superciliosus*, Psu.

Table S6. Singular values and variations explained by first six CVs.

Canonical variants	Eigenvalues	% variance	% Cumulative variance
CV1	18.79998172	35.704	35.704
CV2	6.96039128	13.219	48.923
CV3	6.53344047	12.408	61.331
CV4	4.00945294	7.615	68.946
CV5	3.43507976	6.524	75.469
CV6	2.69705822	5.122	80.591

Table S7. The estimates of evolutionary information for each lineage.

Group	Species	Mean dN	Mean dS	Mean dN/dS
**Total genes**	*P. humilis*	0.003	0.016	0.268
(n=1,873)	*P. major*	0.005	0.021	0.259
	G. fortis	0.012	0.051	0.241
	S. canaria	0.009	0.045	0.228
	C. brachyrhynchos	0.017	0.078	0.236
**Bone formation genes**	*P. humilis*	0.002	0.015	0.171
(n=49)	*P. major*	0.008	0.030	0.205
	G. fortis	0.015	0.059	0.204
	S. canaria	0.007	0.044	0.154
	C. brachyrhynchos	0.013	0.081	0.176

Table S8. Wilcoxon rank sum tests for dN/dS ratios.

Group	Hypothesis tested	*W*	*P*
**Total genes**	*P. humilis* > dN/dS *P. major*	1,780,827	0.419
(n=1,873)	*P. humilis* > dN/dS *G. fortis*	1,714,294	0.230
	P. humilis > dN/dS *S. canaria*	1,818,576	0.051
	P. humilis > dN/dS *C. brachyrhynchos*	1,706,606	0.152
**Bone formation genes**	*P. humilis* < dN/dS *P. major*	1,077	0.382
(n=49)	*P. humilis* < dN/dS *G. fortis*	996	0.148
	P. humilis > dN/dS *S. canaria*	1,214.5	0.924
	P. humilis < dN/dS *C. brachyrhynchos*	1,065.5	0.339

W is the sum of the ranks of the observations. *P* is the value of statistical significance.

Table S9. Top 20 enriched GO Terms for the accelerated 49 genes in Ground Tit.

GO ID	GO Terms	Gene Counts
GO:0015030	Cajal body	2
GO:0006281	DNA repair	4
GO:0006259	DNA metabolic process	5
GO:0006302	Double-strand break repair	3
GO:0000781	Chromosome, telomeric region	2
GO:0005654	Nucleoplasm	5
GO:0048145	Regulation of fibroblast proliferation	2
GO:0000723	Telomere maintenance	2
GO:0048144	Fibroblast proliferation	2
GO:0032200	Telomere organization	2
GO:0006310	DNA recombination	3
GO:0006974	Cellular response to DNA damage stimulus	4
GO:0033554	Cellular response to stress	5
GO:0000724	Double-strand break repair via homologous recombination	2
GO:0000725	Recombinational repair	2
GO:0044428	Nuclear part	6
GO:0031981	Nuclear lumen	5
GO:0006950	Response to stress	6
GO:0016829	Lyase activity	2
GO:0098687	Chromosomal region	2

Table S10. Identified functional categories related to bone morphogenesis.

GO ID	GO Terms	Gene Counts
GO:0001501	Skeletal system development	22
GO:0001502	Cartilage condensation	1
GO:0001649	Osteoblast differentiation	6
GO:0001958	Endochondral ossification	1
GO:0002062	Chondrocyte differentiation	4
GO:0002063	Chondrocyte development	1
GO:0016055	Wnt signaling pathway	16
GO:0030111	Regulation of Wnt signaling pathway	10
GO:0030177	Positive regulation of Wnt signaling pathway	6
GO:0030178	Negative regulation of Wnt signaling pathway	6
GO:0030282	Bone mineralization	4
GO:0030316	Osteoclast differentiation	2
GO:0030500	Regulation of bone mineralization	2
GO:0030501	Positive regulation of bone mineralization	1
GO:0032330	Regulation of chondrocyte differentiation	3
GO:0032331	Negative regulation of chondrocyte differentiation	2
GO:0035567	Non-canonical Wnt signaling pathway	3
GO:0045453	Bone resorption	1
GO:0045667	Regulation of osteoblast differentiation	3
GO:0045668	Negative regulation of osteoblast differentiation	1
GO:0045669	Positive regulation of osteoblast differentiation	2
GO:0046849	Bone remodeling	4
GO:0048704	Embryonic skeletal system morphogenesis	1
GO:0048705	Skeletal system morphogenesis	7
GO:0048706	Embryonic skeletal system development	1
GO:0051216	Cartilage development	7
GO:0060070	Canonical Wnt signaling pathway	10
GO:0060071	Wnt signaling pathway, planar cell polarity pathway	3
GO:0060324	Face development	2
GO:0060346	Bone trabecula formation	1
GO:0060348	Bone development	7
GO:0060349	Bone morphogenesis	1
GO:0060350	Endochondral bone morphogenesis	1
GO:0060560	Developmental growth involved in morphogenesis	5
GO:0060828	Regulation of canonical Wnt signaling pathway	10
GO:0061035	Regulation of cartilage development	3
GO:0061037	Negative regulation of cartilage development	2
GO:0061430	Bone trabecula morphogenesis	1
GO:0098751	Bone cell development	2

Table S11. Descriptions and expression patterns of 26 differentially expressed genes involving in the five pathways.

Pathways	Genes	Descriptions	DESeq		edgeR		NOISeq	
			Log2FC	*P*-adj	Log2FC	*P*-adj	Log2FC	*P*-adj
Wnt	*WIF1**	*Wnt inhibitory factor 1*	-1.39	8.01E-10	-1.38	1.22E-11	-2.97	8.87E-06
	*FRZB**	*Secreted frizzled-related protein 3*	-1.52	1.38E-13	-1.51	1.1E-18	-1.08	0.000347
	*PRICKLE1**	*Prickle-like protein 1*	-1.53	4.51E-13	-1.52	2.42E-17	-1.89	2.75E-06
	*WNT5A**	*Protein Wnt-5a*	-2.81	4.43E-22	-2.80	3.29E-53	-1.63	0
	*DAAM2**	*Disheveled-associated activator of morphogenesis 2*	-1.42	3.46E-07	-1.42	3.05E-10	-2.60	0.000347
	*CCND1**	*G1/S-specific cyclin-D1*	-1.85	8.22E-19	-1.84	3.28E-18	-1.51	0.000418
	*PRKACB**	*cAMP-dependent protein kinase catalytic subunit beta*	-2.19	5.77E-26	-2.18	1.22E-35	-1.10	0.000497
	*NFATC3**	*Nuclear factor of activated T-cells, cytoplasmic 3*	-2.16	2.09E-24	-2.15	7.12E-20	-2.13	9.47E-05
	*FZD4**	*Frizzled-4*	-3.11	1.4E-47	-3.11	4.99E-49	-2.43	0.000154
	*LEF1**	*Lymphoid enhancer-binding factor 1*	-1.29	3.72E-10	-1.28	5.98E-12	-1.24	0.000177
MAPK/FGF	*FGF13**	*Fibroblast growth factor 13*	4.14	1.06E-68	4.15	9.41E-76	1.61	0.000421
	*FGF17**	*Fibroblast growth factor 17*	5.83	1.14E-15	6.12	9.09E-53	6.91	0
	*STK3**	*Serine/threonine-protein kinase 38*	-2.14	1.25E-14	-2.14	2.16E-17	-1.80	0.000464
	MAP3K3	*Mitogen-activated protein kinase kinase kinase 3*	-2.71	6.17E-26	-2.70	1.2E-29	-3.01	9.21E-05
	MAP2K3	*Mitogen-activated protein kinase kinase 3*	-1.88	1.26E-14	-1.87	4.81E-17	-1.94	0.00057
	HSPA2	*Heat shock-related 70 kDa protein 2*	-1.10	1.97E-07	-1.10	5.16E-08	-1.76	0.000154
	RASGRP1	*RAS guanyl-releasing protein 1*	-1.51	3.61E-05	-1.50	7.3E-08	-3.49	0.000383
	CACNG5	*Voltage-dependent calcium channel gamma-5 subunit*	-3.98	3.92E-07	-3.97	4.75E-31	-3.02	0.000487
	PLA2G4B	*Cytosolic phospholipase A2 beta*	5.12	4.48E-31	5.13	7E-95	3.08	0.000559
OCD	*ITGB3**	*Integrin beta-3*	-3.02	4.17E-27	-3.01	3.19E-28	-3.08	8.57E-05
	*NFATC1**	*Nuclear factor of activated T-cells, cytoplasmic 1*	-2.45	1.87E-28	-2.45	6.73E-34	-2.76	1.6E-08
	FOSL2	*Fos-related antigen 2*	-4.70	3.19E-54	-4.69	3.85E-56	-3.31	0.000628
Notch	*HES5**	*Transcription factor HES-5*	3.13	1.97E-06	3.14	1.93E-31	2.77	0.000311
Calcium	*CACNA1G**	*Voltage-dependent T-type calcium channel subunit alpha-1G*	-1.40	2.66E-11	-1.40	1.32E-14	-1.77	0.000579
	PLCB4	*1-phosphatidylinositol 4,5-bisphosphate phosphodiesterase beta-4*	-1.46	7.1E-10	-1.45	1.12E-11	-1.71	0.000533
	ADCY2	*Adenylate cyclase type 2*	-1.42	3.63E-11	-1.41	5.08E-14	-2.07	0.000631

"*" marked the 17 genes related to bone development and morphogenesis. Log2FC is the transformation of the fold change in expression levels. *P*-adj is the adjusted *P*-value using FDR. In NOISeq, *P*-adj is

considered equivalent to 1 − q_{NOISeq}.

Table S12. Function categories annotated for the 17 differential genes.

Rank	GO ID	GO Terms	Genes
1	GO:0051216	Cartilage development	*HES5*, *FRZB*, *WNT5A*
2	GO:0009653	Anatomical structure morphogenesis	*FGF13*, *FGF17*, *WNT5A*, *NFATC1*, *PRICKLE1*, *ITGB3*, *NFATC3*, *FZD4*, *LEF1*, *HES5*, *FRZB*, *STK3*, *PRKACB*, *CACNA1G*
3	GO:0048729	Tissue morphogenesis	*PRICKLE1*, *ITGB3*, *NFATC3*, *LEF1*, *HES5*, *FRZB*, *WNT5A*, *STK3*, *PRKACB*
4	GO:0061448	Connective tissue development	*HES5*, *FRZB*, *WNT5A*
9	GO:0009887	Organ morphogenesis	*HES5*, *FRZB*, *WNT5A*, *LEF1*
11	GO:0001501	Skeletal system development	*HES5*, *FRZB*, *WNT5A*
15	GO:0009888	Tissue development	*WNT5A*, *NFATC1*, *PRICKLE1*, *ITGB3*, *NFATC3*, *FZD4*, *LEF1*, *HES5*, *FRZB*, *CCND1*, *STK3*, *PRKACB*
20	GO:0048598	Embryonic morphogenesis	*WNT5A*, *PRICKLE1*, *ITGB3*, *LEF1*, *HES5*, *FRZB*, *STK3*, *PRKACB*
37	GO:0007275	Multicellular organismal development	*FGF13*, *FGF17*, *WIF1*, *WNT5A*, *NFATC1*, *PRICKLE1*, *ITGB3*, *NFATC3*, *FZD4*, *LEF1*, *HES5*, *FRZB*, *DAAM2*, *CCND1*, *STK3*, *PRKACB*, *CACNA1G*
38	GO:0061035	Regulation of cartilage development	*FRZB*

The 17 differential genes were correlated to bone development and morphogenesis and were clustered in TOP 50 GO terms. Please see the descriptions for these genes in Table S11.

Table S13. Several known genes that account for the evolution of beak morphology and their expression patterns in developmental beaks of the Ground Tit and the Great Tit.

**Genes**	***P. humilis***	***P. major***	**DESeq**		**edgeR**		**NOISeq**	
			**Log2FC**	***P*-adj**	**Log2FC**	***P*-adj**	**Log2FC**	***P*-adj**
BMP4 [4, 5]	1,568.77	1,390.41	0.17	0.743005	0.18	0.502495	-0.60	0.012492
TGFBR2 [6, 7]	--	--	--	--	--	--	--	--
TGFB2 [6, 7]	--	--	--	--	--	--	--	--
CALM1 [8]	20,878.27	12,678.06	0.72	0.000313	0.73	1.36E-05	-0.46	0.003166
DKK3 [6, 7]	1,168.567	1,035.92	0.17	0.513693	0.18	0.414891	0.03	0.997572
CTNNB1 [6, 7]	39,581.96	38,535.45	0.04	0.852479	0.05	0.811193	-0.79	6.00E-15
FZD1 [9]	--	--	--	--	--	--	--	--
FGF8 [10-14]	--	--	--	--	--	--	--	--
SHH [10, 12, 13, 15]	--	--	--	--	--	--	--	--
IHH [6]	--	--	--	--	--	--	--	--
RALDH2 [16]	815.77	93.97	3.12	2.84E-06	3.13	8.46E-21	0.46	0.378729
RALDH3 [16]	--	--	--	--	--	--	--	--
MSX1 [17, 18]	8,606.96	9,667.24	-0.17	0.647613	-0.16	0.504094	-0.34	0.066404

Log2FC is the transformation of the fold change in expression levels. *P*-adj is the adjusted *P*-value using FDR. In NOISeq, *P*-adj is considered equivalent to $1 - q_{NOISeq}$. “--” represents genes excluded from the orthologous gene set.

Table S14. Measurements of beak size (length, width, depth) for 13 parid species.

**Species**	**Sample size**	**Length (Mean±SD)**	**Width (Mean±SD)**	**Depth (Mean±SD)**
Cyanistes cyanus	11	3.120±0.212	1.687±0.103	1.995±0.093
Lophophanes dichrous	16	3.170±0.251	1.578±0.083	1.579±0.103
Melanochlora sultanea	10	3.236±0.191	1.689±0.090	1.889±0.118
Parus humilis	39	5.749±0.506	1.717±0.112	1.838±0.102
Parus major	68	3.190±0.205	1.750±0.072	1.805±0.099
Parus monticolus	39	3.142±0.181	1.787±0.093	1.722±0.115
Parus spilonotus	17	3.392±0.242	1.830±0.148	1.923±0.104
Periparus ater	18	3.574±0.246	1.605±0.137	1.628±0.164
Periparus rubidiventris	22	3.581±0.273	1.758±0.152	1.880±0.141
Periparus venustulus	35	3.203±0.236	1.776±0.142	1.671±0.221
Poecile montanus	22	3.386±0.249	1.799±0.106	1.775±0.127
Poecile palustris	40	3.262±0.235	1.781±0.107	1.853±0.123
Poecile superciliosus	12	3.324±0.156	1.723±0.108	2.027±0.135
Medians	22	3.262	1.750	1.838

Mean±SD: average value of measurement ± standard deviation of measurement.

Table S15. Wilcoxon rank sum test on differences between males and females within species.

Species	Length		Width		Depth		CV1		Centroid size	
	W	***P***	***W***	***P***	***W***	***P***	***W***	***P***	***W***	***P***
Cyanistes cyanus							18	0.53	6	0.16
Lophophanes dichrous	42	0.22	44.5	0.13	24.5	0.59	12	0.06	39	0.47
Parus major	376.5	0.30	314	1	283	0.62	541	0.84	604	0.64
Periparus ater	24.5	1	24.5	1	24.5	1	40.5	1	40.5	1
Poecile montanus	22	0.53	22	0.53	22	0.53	14	0.41	6	0.41

The results of the test showed no differences in beak size and shape between sexes. Length, width and depth measurements are standardized by the cube root of body mass. The absent species in this table have all male samples. *W* is the sum of the ranks of the observations. *P* is the value of statistical significance.

Table S16. Comparison of incubation periods and embryogenesis of a few bird species.

Organisms	Incubation period	Embryonic days (E) matching with HH28/29	Key identification features at HH28/29
Chicken [19]	20-21 d	E5.5-6	
Japanese Quail [20]	~16.5 d	E5.5-6	•Beak outgrowth is distinct and
Society Finch [21]	~17 d	E6.5	more prominent;
Zebra Finch [22]	~11 d	E6-6.5	•Digits and toes distinct;
Darwin's finches [4]	11-12 d	E6	•Wing bent in elbow visible;
Ground Tit	14-16 d	ca. E6-6.5	•No egg tooth apparent;
Great Tit	12-15 d	ca. E6-6.5	

By comparing the stage system of multiple species, we finally did staging for the Ground Tit and Great Tit. HH, Hamburger and Hamilton stage.

Table S17. The statistics and quality assessment for raw data and clean data after filtration.

Vouch number	Sample	RIN	Raw Reads	Clean Reads	Clean Base (bp)	Effective Rate (%)	Error Rate (%)	Q20 (%)	Q30 (%)	GC Content (%)
18765	PhuB1	6.8	24,228,033	21,689,370	4,337,874,000	89.52	0.01; 0.01	99.34; 99.29	95.99; 95.63	47; 47
18771	PhuB2	8.6	24,895,764	22,209,206	4,441,841,200	89.21	0.01; 0.01	99.34; 99.28	95.97; 95.55	47; 47
18743	PmaB1	7.2	31,747,107	28,570,866	5,714,173,200	89.99	0.01; 0.01	99.36; 99.31	96.05; 95.71	47; 47
18748	PmaB2	7.2	28,685,318	25,599,330	5,119,866,000	89.24	0.01; 0.01	99.34; 99.28	95.97; 95.56	47; 47

PhuB1, replicate 1 of Ground Tit beak; PhuB2, replicate 2 of Ground Tit beak; PmaB1, replicate 1 of Great Tit beak; PmaB2, replicate 2 of Great Tit beak. RIN is RNA Integrity Number. Effective rate represents the ratio of clean reads to raw reads. Q20 is the probability of the bases that have Phred quality score > 20. Q30 is the probability of the bases that have Phred quality score > 30. The two values in last four columns correspond to the assessed values of pair end data.

Table S18. RT-qPCR primers used for the cDNA of both species.

Symbol	Sequence (5’-3’)
FGF13-F	GGGGTTGGTATTTGGGTCTT
FGF13-R	CTCCTGAAACGCTTCTGCTC
FGF17-F	CTGGTACATGGCCTTCACCC
FGF17-R	GCCCACGAACTCGAACTGC
FRZB-F	ATGGGCTACGAGGACGAGG
FRZB-R	CCAAGGTGGCGGAGTTTCT
HES5-F	ACAAGAACCCAGAGCAGG
HES5-R	GAAACACGCAGGGAGACA
ITGB3-F	CAAGTGCGTCAAGTGGAA
ITGB3-R	GATGCGTAGGTTGCTGGT
NFATC1-F	GGAAGTGCCAAGGTCTA
NFATC1-R	GAGGGACTGTTGGGATAG
PRICKLE1-F	TGTGCCTGTACTGAGACG
PRICKLE1-R	AAATGGTAAACACGCCTA
WIF1-F	CCCGTTCACCCATGACTTTA
WIF1-R	TCAGCCATTATGCCTTTATCC
GAPDH-F	AACCAGCCAAGTACGATGACAT
GAPDH-R	CCATCAGCAGCAGCCTTCA

F means forward strand, while R means reverse strand.

References

1. ESRI. ArcGIS Desktop: Release 9.3. Redlands, CA: Environmental Systems Research Institute. 2008.
2. Blomberg SP, Garland T, Ives AR. Testing for phylogenetic signal in comparative data: Behavioral traits are more labile. Evolution. 2003;57:717-745.
3. Pagel M. Inferring the historical patterns of biological evolution. Nature. 1999;401:877-884.
4. Abzhanov A, Protas M, Grant BR, Grant PR, Tabin CJ. Bmp4 and morphological variation of beaks in Darwin's finches. Science. 2004;305:1462-1465.
5. Wu P, Jiang TX, Suksaweang S, Widelitz RB, Chuong CM. Molecular shaping of the beak. Science. 2004;305:1465-1466.
6. Mallarino R, Campas O, Fritz JA, Burns KJ, Weeks OG, Brenner MP, Abzhanov A. Closely related bird species demonstrate flexibility between beak morphology and underlying developmental programs. Proc Natl Acad Sci U S A. 2012;109:16222-16227.
7. Mallarino R, Grant PR, Grant BR, Herrel A, Kuo WP, Abzhanov A. Two developmental modules establish 3D beak-shape variation in Darwin's finches. Proc Natl Acad Sci U S A. 2011;108:4057-4062.
8. Abzhanov A, Kuo WP, Hartmann C, Grant BR, Grant PR, Tabin CJ. The calmodulin pathway and evolution of elongated beak morphology in Darwin's finches. Nature. 2006;442:563-567.
9. Brugmann SA, Powder KE, Young NM, Goodnough LH, Hahn SM, James AW, Helms JA, Lovett M. Comparative gene expression analysis of avian embryonic facial structures reveals new candidates for human craniofacial disorders. Hum Mol Genet. 2010;19:920-930.
10. Abzhanov A, Tabin CJ. Shh and Fgf8 act synergistically to drive cartilage outgrowth during cranial development. Dev Biol. 2004;273:134-148.
11. Szabo-Rogers HL, Geetha-Loganathan P, Nimmagadda S, Fu KK, Richman JM. FGF

signals from the nasal pit are necessary for normal facial morphogenesis. Dev Biol. 2008;318:289-302.

12. Wu P, Jiang TX, Shen JY, Widelitz RB, Chuong CM. Morphoregulation of avian beaks: comparative mapping of growth zone activities and morphological evolution. Dev Dyn. 2006;235:1400-1412.

13. Bhullar BA, Morris ZS, Sefton EM, Tok A, Tokita M, Namkoong B, Camacho J, Burnham DA, Abzhanov A. A molecular mechanism for the origin of a key evolutionary innovation, the bird beak and palate, revealed by an integrative approach to major transitions in vertebrate history. Evolution. 2015;69:1665-1677.

14. Mina M, Wang YH, Ivanisevic AM, Upholt WB, Rodgers B. Region- and stage-specific effects of FGFs and BMPs in chick mandibular morphogenesis. Dev Dyn. 2002;223:333-352.

15. Hu D, Young NM, Li X, Xu YH, Hallgrimsson B, Marcucio RS. A dynamic Shh expression pattern, regulated by SHH and BMP signaling, coordinates fusion of primordia in the amniote face. Development. 2015;142:567-574.

16. Song Y, Hui JN, Fu KK, Richman JM. Control of retinoic acid synthesis and FGF expression in the nasal pit is required to pattern the craniofacial skeleton. Dev Biol. 2004;276:313-329.

17. Medio M, Yeh E, Popelut A, Babajko S, Berdal A, Helms JA. Wnt/beta-catenin signaling and Msx1 promote outgrowth of the maxillary prominences. Front Physiol. 2012;3:375.

18. Mina M, Gluhak J, Upholt WB, Kollar EJ, Rogers B. Experimental analysis of Msx-1 and Msx-2 gene expression during chick mandibular morphogenesis. Dev Dyn. 1995;202:195-214.

19. Hamburger V, Hamilton HL. A series of normal stages in the development of the chick embryo. J Morphol. 1951;88:49-92.

20. Ainsworth SJ, Stanley RL, Evans DJR. Developmental stages of the Japanese quail. J Anat. 2010;216:3-15.

21. Yamasaki M, Tonosaki A. Developmental stages of the Society Finch, *Lonchura striata* var. domestica. Dev Growth Differ. 1988;30:515-542.

22. Murray JR, Varian-Ramos CW, Welch ZS, Saha MS. Embryological staging of the Zebra Finch, *Taeniopygia guttata*. J Morphol. 2013;274:1090-1110.
